# Dragmacidin G, a Bioactive Bis-Indole Alkaloid from a Deep-Water Sponge of the Genus *Spongosorites*

**DOI:** 10.3390/md15010016

**Published:** 2017-01-11

**Authors:** Amy E. Wright, K. Brian Killday, Debopam Chakrabarti, Esther A. Guzmán, Dedra Harmody, Peter J. McCarthy, Tara Pitts, Shirley A. Pomponi, John K. Reed, Bracken F. Roberts, Carolina Rodrigues Felix, Kyle H. Rohde

**Affiliations:** 1 Harbor Branch Oceanographic Institute, Florida Atlantic University, Fort Pierce, FL 34946, USA; eguzman9@fau.edu (E.A.G.); dharmody@hboi.fau.edu (D.H.); pmccart5@fau.edu (P.J.M.); tpitts3@fau.edu (T.P.); spomponi@fau.edu (S.A.P.); jreed12@fau.edu (J.K.R.); 2 Bruker Biospin Corporation, Billerica, MA 01821, USA; Brian.Killday@bruker.com; 3 Burnett School of Biomedical Sciences, University of Central Florida, Orlando, FL 32827, USA; Debopam.Chakrabarti@ucf.edu (D.C.); bracken.roberts@knights.ucf.edu (B.F.R.); carolinarodriguesfelix@knights.ucf.edu (C.R.F.); Kyle.Rohde@ucf.edu (K.H.R.)

**Keywords:** bis(indole) alkaloid, *Spongosorites*, antibacterial, pancreatic cancer

## Abstract

A deep-water sponge of the genus *Spongosorites* has yielded a bis-indole alkaloid which we have named dragmacidin G. Dragmacidin G was first reported by us in the patent literature and has recently been reported by Hitora et al. from a sponge of the genus *Lipastrotheya*. Dragmacidin G is the first in this series of compounds to have a pyrazine ring linking the two indole rings. It also has a rare *N*-(2-mercaptoethyl)-guanidine side chain. Dragmacidin G shows a broad spectrum of biological activity including inhibition of methicillin-resistant *Staphylococcus aureus*, *Mycobacterium tuberculosis*, *Plasmodium falciparum,* and a panel of pancreatic cancer cell lines.

## 1. Introduction

Sponges of the genus *Spongosorites* have been a source of a number of biologically active bis-indole alkaloids including the topsentins [1,2,3], nortopsentins [4,5], hamacanthins [2,6], and dragmacidins D–F [7,8,9]. They have been reported to have a variety of activities including antibacterial, antiviral, antifungal, antiplasmodial, cytotoxic, and anti-inflammatory activities. As part of a study to provide larger quantities of the topsentins for evaluation as anti-inflammatory agents, a sponge of the genus *Spongosorites* was collected using the *Johnson-Sea-Link* manned submersible. This specimen was extracted and chromatographed by vacuum column chromatography and yielded topsentin and bromotopsentin as major components. Interestingly, this specimen also contained a major compound not previously observed in other specimens. This compound was retained on C-18 reversed-phase packing when eluted with mixtures of CH_3_CN:H_2_O or 100% CH_3_CN but rapidly elutes from the column as a dark red band with the addition of 0.1% trifluoroacetic acid (TFA) to the CH_3_CN:H_2_O eluent. NMR analysis of this fraction indicated that it was primarily one major compound and had resonances characteristic of a bis-indole alkaloid similar to those reported previously from *Spongosorites*. Fractions from this separation were added to the HBOI-enriched fraction library and screened in a number of assays including growth inhibition of the drug-resistant human pathogenic bacteria, methicillin-resistant *Staphylococcus aureus* (MRSA); growth inhibition of the causative agent for tuberculosis, *Mycobacterium tuberculosis*; and growth inhibition of the drug resistant DD2 strain of *Plasmodium falciparum.* The highly enriched fraction containing the new compound showed activity in all three assays and therefore was further purified to obtain pure dragmacidin G, 1, enabling its structure elucidation and biological testing. We first reported this structure in a patent [10], and it has very recently been reported by Hitora et al. from a sponge of the genus *Lipastrotethya* [11]. The following description adds data supporting the structure assignment to that included in the Hitora paper as well as eliminates an alternative pyrazinine structure that was not considered in that work. We also report on the substantial antibacterial activity of the compound, which is greater than 10-fold more potent than other members of the bis-indoles while retaining selectivity towards bacterial over mammalian cells.

## 2. Results

### 2.1. Isolation of ***1***

**Figure 1:**
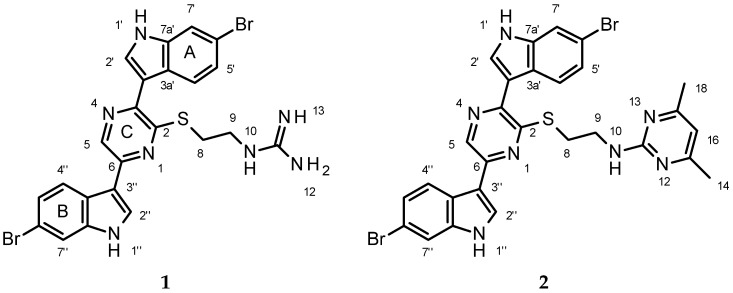
Structure of Dragmacidin G and its pyrimidine derivative. A is the first indole moiety, B is the second indole moiety and C is the pyrazine.

The sponge (See Figure S1) is an unidentified species of *Spongosorites* (Phylum Porifera, Class Demospongiae, Order Suberitida, Family Halichondriidae) [12]. It was collected from Long Island, Bahamas (23°41.12′ N, 75°22.18′ W), using the Johnson-Sea-Link I manned submersible at a depth of 630 m. The compound was purified using repeated chromatography on C-18 reversed phase stationary phases to yield **1** as a reddish brown glass (Figure 1).

### 2.2. Structure Elucidation of ***1***

Inspection of the ^13^C NMR spectrum coupled with HRMS data and isotopologue comparison suggested a molecular formula of C_23_H_19_Br_2_N_7_S for **1** [(M + H^+^) *m*/*z* observed 583.98688, calcd. 583.986761 Δ = −0.12 mmu)] requiring 17 degrees of unsaturation. Isotopologue matching algorithms support the presence of sulfur in the molecule (see Figure S2). The ^13^C NMR spectrum (See Figure S4) has resonances for 21 sp^2^ hybridized carbons observed between δ_C_ 156.8 and 111.8 ppm and 2 sp^3^ hybridized carbons observed at δ_C_ 40.2 and 28.7 ppm. The ^1^H NMR spectrum (See Figure S3) coupled with the edited *g*-HSQC (see Figures S5 and S6) and ^1^H-^15^N HMBC spectra shows the presence of three protons attached to nitrogen (δ_H_, 11.95, 11.82, and 7.93), nine olefinic methine protons between δ_H_ 8.98 and 7.25 ppm, and four methylene protons observed as overlapping resonances between δ_H_ 3.58 and 3.53 ppm.

The ^1^H NMR spectrum coupled with the edited *g*-HSQC spectrum and a series of HMBC experiments support the presence of two 6-substituted-indol-3-yl ring systems in **1**. The spectra for **1** have substantial overlap for the indole resonances, but careful analysis of the HSQC and HMBC spectra (including the ^1^H-^13^C HMBC (see Figures S7–S11), a band selective ^1^H-^13^C HMBC (See Figures S12–S16) and the ^1^H-^15^N HMBC (see Figure S17)) allowed for assignment of all atoms in the indol-3-yl rings (See Figure S25).

For the first indole labeled A in Figure 1, residual one bond couplings observed in the ^1^H-^15^N *g*-HMBC spectrum assign the proton observed at 11.82 (H-1′) as being attached to a nitrogen observed at δ_N_ 136. Correlations in the 2D-COSY spectrum (See Figures S18–S21) show that this proton is coupled to the proton observed at δ_H_ 8.08 (H-2′). A 1,2,4-trisubstituted aromatic ring as found in 6-substituted indoles was suggested from the 2D-*g*-COSY spectrum in which the resonance observed at 8.10 (d *J =* 8.9 Hz) shows coupling to a proton observed at 7.25 (dd *J =* 8.9, 1.4 Hz), which in turn shows coupling to a proton observed at 7.70 (d *J =* 1.4 Hz). This latter proton shows a correlation in the ^1^H-^15^N *g*-HMBC to the nitrogen observed at δ_N_ 136 assigning it as H-7′ of the indole ring. The chemical shifts of a number of carbons are very close, but most could be distinguished in a band selective ^1^H-^13^C *g*-HMBC experiment selected for carbon resonances between δ_C_ 110 and 160 ppm. All correlations predicted for a 6-subsituted-3-indolyl functionality were observed (Table 1, Figure S25). The chemical shift of C-6′ is consistent with bromine substitution (δ_C_ 115.37) as observed in the topsentins, dragmacidins, and related compounds; therefore, the bromine was assigned to this position.

Similar arguments led to the assignment of the second 6-bromo-indol-3-yl moiety in **1** (labeled B in Figure 1). For the second indol-3yl ring, residual one bond couplings observed in the ^1^H-^15^N *g*-HMBC spectrum assign the proton observed at 11.95 (H-1″) as being attached to the nitrogen observed at δ_N_ 138. Correlations in the 2D-*g*-COSY spectrum show that this proton is coupled to the proton observed at δ_H_ 8.33 (H-2″). A 1,2,4-substituted aromatic ring is suggested by the spin system observed in the 2D-*g*-COSY spectrum in which the resonance observed at 8.22 (d *J =* 8.9 Hz) shows coupling to a proton observed at 7.31 (dd *J =* 8.9, 1.4 Hz), which in turn shows coupling to a proton observed at 7.71 (d *J =* 1.4 Hz). This latter proton shows a correlation in the ^1^H-^15^N *g*-HMBC to the nitrogen at δ_N_ 138 assigning it as H-7″ of the indole ring. Once again, the chemical shift of C-6″ was consistent with bromine substitution (δ_C_ 114.78); therefore, the second bromine was assigned to this position. As with the first indole ring, all expected correlations were observed in the HMBC spectra supporting the assignment of a second 6-bromo-indol-3-yl ring in **1** (Table 1, Figure S4).

The presence of an *N*-(2-mercaptoethyl) guanidine moiety in **1** was suggested by the data that follows. A broad four-proton multiplet was observed between δ_H_ 3.58 and 3.53 ppm in the ^1^H NMR spectrum. Careful inspection of the edited *g*-HSQC, ^1^H-^13^C *g*-HMBC, and ^1^H-^15^N *g*-HMBC allow for the assignment of one methylene group at δ_H_ 3.56 attached to a carbon observed at δ_C_ 28.7 and a second methylene group observed at δ_H_ 3.54 ppm attached to a carbon observed at δ_C_ 40.2. These ^1^H resonances are too close to each other to detect scalar coupling between them, but each proton shows a correlation to the other carbon in the ^1^H-^13^C *g*-HMBC spectrum, suggesting the presence of ethyl functionality. Correlations observed in the 2D-*g*-COSY spectrum between the protons observed at δ_H_ 3.54 ppm and δ_H_ 7.93 (bt *J =* 4.8 Hz) extend this chain. The proton observed at δ_H_ 7.93 is attached to a nitrogen observed at δ_N_ 80 based upon residual one bond coupling observed in the ^1^H-^15^N *g*-HMBC experiment. The ^1^H-^13^C *g*-HMBC shows a correlation from the methylene group observed at δ_H_ 3.54 to the quaternary carbon observed at 156.8 ppm, suggesting that this carbon is attached to the nitrogen (N-10). The proton resonance observed at 7.93 has long range coupling to an additional nitrogen resonance observed at 72 ppm in the ^1^H-^15^N *g*-HMBC spectrum. The presence of an additional nitrogen resonance as well as the chemical shift of 156.8 is consistent with the presence of an *N*-ethyl guanidine functionality in **1**. The molecular formula of **1** calls for the presence of a sulfur atom. A search of the literature reports the presence of an *N*-(2-mercaptoethyl)-guanidine unit in phloeodictine B isolated from the sponge *Phloeodictyon* sp. [13]. Comparison of the NMR data to that of **1** shows a fairly close correlation (**1**: δ_H_ 3.54, 3.56, 7.93; δ_C_ 28.7, 40.2, 156.8; phloeodictine B δ_H_ 3.40, 3.60, 8.25; δ_C_ 31.7, 40.9, 158.6). The presence of guanidine functionality was confirmed by reaction of **1** with 2,4-pentanedione to form the 3,5-dimethyl pyrimidine derivative **2** (Figure 1).

The remaining atoms in the molecule are an olefinic methine group (δ_H_ 8.98 s, δ_C_ 135.4), three quaternary olefinic carbons (δ_C_ 150.1, 145.9, 143.3), and two nitrogen atoms (δ_N_ 324, 299) whose chemical shifts are consistent with being part of a heteroaromatic ring [14]. Correlations observed in the ^1^H-^13^C *g*-HMBC spectrum from H-2′ of indole “A” to the non-protonated olefinic carbon observed at δ_C_ 143.3 suggests the attachment of the “A” indole to this carbon. Similarly, a correlation observed in the ^1^H-^13^C *g*-HMBC spectrum between H-2″ of the “B” ring indole and the carbon observed at δ_C_ 145.9 suggests the attachment of the “B” indole to this carbon.

**Figure 2:**
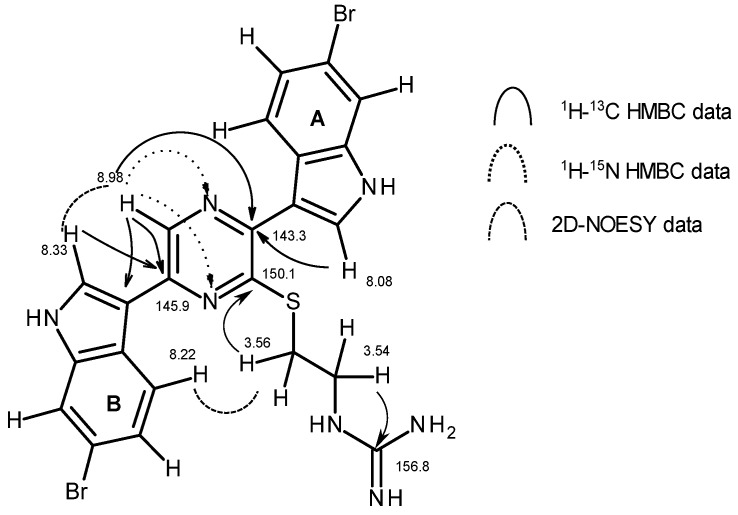
Key HMBC and NOESY correlations supporting the structure assignment of **1**.

The heteroaromatic methine proton observed at δ_H_ 8.98 has a number of correlations in both the ^1^H-^13^C and ^1^H-^15^N *g*-HMBC spectra that allow for construction of the “C” ring (Figure 2). Correlations observed in the ^1^H-^13^C *g*-HMBC spectrum between the methine proton observed at δ_H_ 8.98 and both C-3″ of the “B” indole (δ_C_ 112.6), and the carbon observed at 145.9 suggested the attachment of the methine group (C-5) to the carbon observed at 145.9 (C-6), which is in turn attached to C-3″. The ^1^J_CH_ coupling constant for this methine group was observed through residual one bond resonances in the ^1^H-^13^C *g*-HMBC experiment (See Figure S22) and was found to be 183 Hz, which is consistent with substitution of a single nitrogen atom [15]; therefore, a nitrogen substituent was placed as the final substituent on this carbon (C-5). The methine proton observed at δ_H_ 8.98 (H-5) showed a strong correlation in the ^1^H-^13^C HMBC spectrum to the olefinic carbon observed at δ_C_ 143.3, suggesting that this carbon is attached to N-4. The methine proton showed strong correlations in the ^1^H-^15^N *g*-HMBC spectrum to two nitrogens observed at δ_N_ 324 and 299. One of the nitrogens must be N-4, while the other must be attached to the carbon observed at δ_C_ 145.9. These nitrogen chemical shifts are consistent with nitrogens in a pyrazine ring [14]. The final atom to be placed in the molecule is the olefinic carbon observed at δ_C_ 150.1. By default, it has been placed between N-1 and C-3 to form the pyrazine ring. A correlation observed in the ^1^H-^13^C HMBC spectrum between the carbon observed at δ_C_ 150.1 (C-2) and the methylene protons observed at δ_H_ 3.56 allowed for placement of the *N*-(2-mercaptoethyl) guanidine moiety on C-2 through the sulfur atom. The assignment of a pyrazine ring is further supported by the nOe data observed in the 2D-*g*-NOESY spectrum (See Figures S23, S24 and S26). The 2D-*g*-NOESY spectrum shows correlations between both the H-2″ and H-4″ protons of the “B” indole ring with the methine proton observed at δ_H_ 8.98 (H-5). H-4″ also showed a strong correlation with the H_2_-8 methylene group of the *N*-(2-mercaptoethyl)-guanidine moiety. No correlations are observed from these resonances to any resonances in the “A” ring indole. The observed nOes are consistent with what would be expected for the pyrazine structure in which one indole ring (“A”) is not in close proximity to either H-5 or the methylene protons of the ethyl side chain. The latter is due to the near 90 degree angle observed in the C–S–C bond, which places the H_2_-8 methylene protons in proximity to H-4″ of the “B” ring indole.

An alternative structure which was considered would have a pyrazinine ring (1,2 placement of the nitrogen atoms in a six membered ring). To accommodate all of the observed HMBC data (^1^H-^13^C and ^1^H-^15^N) the two indole rings would be on adjacent carbons of the pyrazinine with the *N*-(2-mercaptoethyl) guanidine side chain being placed para to the methine (See Figure S27). This structure was eliminated based upon the observed nOe data in which the 2D-NOESY spectrum shows clear proximity of the “B” indole to the *N*-(2-mercaptoethyl) guanidine side chain, which would not be possible with this structure (Figure S6). Given the placement of the two indoles on adjacent carbons, one might also expect to observe some nOe between protons on the two indole rings, but none is observed. Calculated spectra obtained using ACD’s NMR Predictor software were consistent with the observed data for the pyrazine structure (See Figure S28). One unusual feature was the observation of a weak correlation in the ^1^H-^13^C HMBC spectrum between H-5 and C-2 of the pyrazine (a 1,4 coupling). All possible orientations of a six membered ring would require at least one of the HMBC correlations observed in the ^1^H-^13^C or ^1^H-^15^N HMBC spectra to be a 1,4 coupling. A five-membered ring can be drawn that accommodates all the HMBC data but requires the presence of an N-amino pyrrole ring. Calculated chemical shifts for this ring system are very different than the observed chemical shifts and the *N*-amine nitrogen would not be expected to be at δ_N_ 299 or 324 ppm; therefore, this structure has been ruled out. Taking into account both the agreement with calculated chemical shift values for both ^1^H and ^13^C, and the observed nOes, the final structural unit in **1** is assigned as a pyrazine ring system with the “A” indole ring on carbon 3, the *N*-(2-mercaptoethyl)-guanidine moiety linked through the sulfur atom on C-2, and the “B” indole ring on C-6 (Figure 2).

### 2.3. Biological Activity Observed for ***1***

Dragmacidin G showed activity against *S. aureus* and MRSA with minimum inhibitory concentrations (MICs) of 0.62 μg/mL (1 μM) and 0.62 μg/mL (1 μM), respectively. The anti-mycobacterial activity of the compound was assayed using *M. tuberculosis* CDC1551 carrying the pMV306hsp-LuxG13 integrative plasmid, which provides constitutive expression of the *luxCDABE* operon. Dragmacidin G gave an MIC of 21.0 μM with cytotoxicity of 125 μM against the host J744 macrophage cells (selectivity index = 5.95). An assay against the drug-resistant DD2 strain of *Plasmodium falciparum* showed a modest inhibition with an IC_50_ of 6.4 μM, but was almost equally cytotoxic to the NIH 3T3 mouse fibroblast cell line (IC_50_ = 7.8 μM), indicating no selectivity for the malaria parasite. Dragmacidin G was assayed against a panel of pancreatic cancer cell lines including the PANC-1 human pancreatic carcinoma, MIA PaCa-2 human pancreatic carcinoma, BxPC-3 human pancreatic adenocarcinoma, and ASPC-1 human pancreatic adenocarcinoma with IC_50_ values at 72 h of treatment of 18 ± 0.4 μM, 26 ± 1.4 μM, 14 ± 1.4 μM, and 27 ± 0.8 μM, respectively.

## 3. Discussion

Dragmacidin G has selectivity towards *S aureus* (~10 fold), MRSA (~10 fold), and *M. tuberculosis* (5.95 fold) versus mammalian cells. In vivo efficacy and toxicity evaluation would be needed to determine if it has sufficient selectivity to be used as a new antibacterial agent. Related bis-indole alkaloids in the hamacanthin, and topsentin series along with synthetic analogs have been reported to have antibacterial activity against in vitro MRSA (MICs range from 15.4 to 25.6 μM with a selectivity of >4). These same compounds have been reported to show low nanomolar activity against the bacterial pyruvate kinase [16,17,18]. Dragmacidin G is approximately 15-fold more potent than these other compounds against MRSA. Data is not available on its activity against the bacterial pyruvate kinase. Dragmacidin G is the first in the series to have a pyrazine ring linking the two indole rings. It is also the first in the series to have the *N*-(2-mercaptoethyl) guanidine moiety. Phloeodictine B, which shares the *N*-(2-mercaptoethyl)guanidine moiety, has been reported to have antibacterial activity against *S. aureus*, but phloeodictine A which lacks this unit has broader spectrum antibacterial activity and is slightly more potent against *S. aureus* (2.2 μM for phloeodictine A versus 5.6 μM for phloeodictine B) [13], suggesting that the *N*-(2-mercaptoethyl) guanidine side chain may not be necessary for antibacterial activity in the phloeodictine series of compounds. Its role in dragmacidin G’s activity has not been assessed. The pyrazine linking group is unusual in the bis-indole alkaloids with only one other compound being reported from a natural source. Alocasin A has been isolated from the plant *Alocasia macrorhizza* used in folk medicine in Asia and has a pyrazine ring system linking two 5-hydroxy substituted indole rings [19]. This compound was reported to have very modest antiproliferative activity against the Hep-2 (human throat) and Hep-G2 (hepatocarcinoma) cell lines with IC_50_ values of 151 and 85 μM, respectively. No antibacterial data was reported for alocasin A.

## 4. Materials and Methods

### 4.1. General Experimental Procedures

Reversed-phase C-18 flash chromatography was performed using a Tekedyne Isco Combiflash^®^ RFx4 equipped with PeakTrak software (Version 2.1.19, Teledyne Isco, Lincoln, NE, USA). NMR data for **1** were collected on a JEOL ECA-600 spectrometer (JEOL USA, Peabody, MA, USA) operating at 600.17 MHz for ^1^H, 150.9 MHz for ^13^C, and 60.8 MHz for ^15^N (instrument reference set to liquid NH_3_). The edited-*g*-HSQC spectrum was optimized for 140 Hz, the *g*-HMBC spectrum was optimized for 8 Hz, and the band selective *g*-HMBC experiment was optimized for 8 Hz. Chemical shifts were referenced to solvent, e.g., DMSO-*d*_6_ δ_H_ observed at 2.50 ppm and δ_C_ observed at 39.51 ppm. Chemical shifts for ^15^N were referenced to liquid NH_3_ with long range *J*_H,N_ optimized for 6 Hz. NMR data for **2** were collected on a Bruker AMX 500 operating at 500 MHz for ^1^H and 125 MHz for ^13^C (Bruker Biospin, Billerica, MA, USA). The HRMS spectrum was measured using a JEOL AccuTOF-DART 4G (JEOL USA, Peabody, MA, USA) using a prototype paper spray attachment. Isotope matching was used to confirm the presence of sulfur in the molecule (Figure S2). Calculated NMR spectra were generated using Advanced Chemical Development ChemPredictor Software (Version 11 Advanced Chemical Development, Toronto, ON, Canada).

### 4.2. Biological Material

The sponge is an unidentified species of *Spongosorites* (Phylum Porifera, Class Demospongiae, Order Suberitida, Family Halichondriidae) [12]. It was collected from Long Island, Bahamas (23°41.12′ N, 75°22.18′ W), by the *Johnson-Sea-Link I* manned submersible at a depth of 630 m. It was thickly encrusting (approximately 5 cm thick × 20 cm long), firm in consistency, bright yellow alive, and dark brown in ethanol. Clusters of oscules are visible, scattered along the surface of the sponge. The ectosome is easily detachable. Spicules are oxeas, 400 μm long × 10 μm wide. Encrusted on approximately one-third of the surface is another sponge, white when live, that is an unidentified species in the class Demospongiae, order Haplosclerida [20]. This sponge is approximately 0.5 cm thick, brittle, and with two size classes of oxeas (500 μm long × 15 μm wide and 120 μm long × 7 μm wide). A taxonomic reference sample of both sponges was deposited in the Harbor Branch Oceanographic Museum, catalog number 003:00936.

### 4.3. Isolation of Dragmacidin G *(**1**)*

The sponge specimen was frozen at −20 °C immediately after collection and stored frozen until extracted. The frozen material as described above (255 g) was extracted exhaustively with ethanol by macerating in a Waring blender, filtering off tissue and re-extracting the tissue a total of 5 times (2.5 L total). The extract was concentrated to a dark orange oil (15.9 g) by distillation under reduced pressure. The residue was partitioned between *n*-butanol and H_2_O and after concentration by distillation under reduced pressure yielded 5.16 g of butanol partition and 11.85 g of aqueous partition. A total of 2.64 g of the butanol partition was chromatographed under vacuum column chromatographic conditions on a custom-made C-18 reversed-phase stationary phase [21]. The column used had a volume of 350 mL and was 4 cm in height. The butanol partition was adsorbed onto RP-18 packing and applied to the column as a slurry in water. The column was eluted as a step gradient of 400 mL fractions as follows: Fraction 1, H_2_O; Fraction 2, H_2_O:CH_3_CN (80:20 *v*/*v*); Fraction 3, H_2_O:CH_3_CN (60:40 *v*/*v*); Fraction 4, H_2_O:CH_3_CN (60:40 *v*/*v*); Fraction 5, H_2_O:CH_3_CN (50:50 *v*/*v*); Fraction 6 H_2_O:CH_3_CN (50:50 *v*/*v*); Fraction 7 H_2_O:CH_3_CN (40:60 *v*/*v*); Fraction 8, H_2_O:CH_3_CN (20:80 *v*/*v*); Fraction 9 CH_3_CN; Fraction 10 H_2_O:CH_3_CN:trifluoracetic acid (TFA) (35:65:0.1 *v*/*v*/*v*). Fraction 10 had a total weight of 775.2 mg and a subsample was further separated by medium pressure chromatography using a CombiFlash^®^ Rf 4× flash chromatography system as follows: 75 mg of Fraction 10 was adsorbed onto approximately 2 g of C-18 reversed-phase packing and loaded into a loading column. The column used for the chromatography was a Teledyne Isco 5.5 g RediSep Rf Gold^®^ reversed-phase C18 column. The flow rate for the separation was 18 mL/minute. Solvent A was H_2_O:CH_3_CN:TFA 95:5:0.1 (*v*/*v*/*v*) and Solvent B was CH_3_CN:TFA (100:0.1 *v*/*v*). A step gradient was used in which column volumes were used rather than times to allow for changes in flow due to pressure. CV = 0 to CV = 5 were eluted with 100% A; CV = 5 to CV = 40 were eluted as a linear gradient from 100% A to 100% B; CV = 40 to CV = 48 were eluted with 100% B. The column was then washed with CH_3_OH and CH_2_Cl_2_. Pure dragmacidin G eluted as a large peak between 26 and 28 column volumes. A total of 46.5 mg was obtained. Extrapolating back to wet weight of sponge, dragmacidin G is a major component of the sponge and is present at 0.36% of wet weight of sponge.

### 4.4. Conversion of Dragmacidin G *(**1**)* to Pyrimidine Derivative *(**2**)*

A solution of dragmacidin G (**1**, 40 mg, 0.68 mmol) in pyridine (0.5 mL) and 2,4-pentanedione (0.5 mL) was heated at 125 °C for 20 h in a sealed tube. The solution was evaporated in vacuo and the residue was separated on a silica gel column by using 9:1 chloroform/methanol to afford **2** (26.5 mg, 65% theoretical) as an oil.

*Dragmacidin G* (**1**): red glass; λ_max_ 227, 278, 305 sh, 357 nm; ^1^H and ^13^C NMR (Table 1); HRMS: (M + H^+^) *m*/*z* 583.98688 observed, calcd. for C_23_H_20_Br_2_N_7_S, 583.986761 (Δ = −0.12 mmu).

*Pyrimidine derivative* (***2***): orange oil; ^1^H NMR (DMSO-*d*_6_, 500 MHz) 11.8 bs (H-1″), 11.68 bs (H-1′), 8.92 s (H-5), 8.3 d (*J* = 2.7 Hz; H-2″), 8.26 d (*J* = 8.9 Hz; H-4″), 8.11 d (*J* = 8.9 Hz; H-4′), 8.08 d (*J* = 8.9 Hz; H-2′), 7.67 d (*J* = 2.1 Hz; H-7′), 7.65 d (*J* = 1.4 Hz H-7″), 7.23 dd (*J* = 8.9, 2.1 Hz; H-5′), 7.13 dd (*J* = 8.9, 1.4 Hz; H-5″), 7.12 br (H-10), 6.28 s (H-16), 3.70 m (H_2_-9), 3.56 t (*J* = 6.9 Hz; H_2_-8), 2.1 bs (H_3_-14/18); ^13^C NMR (DMSO-*d*_6_, 125 MHz) 162.0 C (C-11), 160.0 C (C-15), 151.0 C (C-2), 145.6 C (C-6), 143.2 C (C-3), 137.8 C (C-7a″), 137 C (C-7a′), 134.8 CH (C-5), 129.2 CH (C-5″), 127.9 C (C-2′), 127.5 C (C-2″), 125.4 C (C-3a′), 124.0 C (C-3a″), 123.2 CH (C-4′), 122.7 CH (C-5′), 122.7 CH (C-4″), 114.6 C (C-6″), 114.6 C (C-6′), 114.5 CH (C-7′), 114.3 CH (C-7″), 112.6 C (C-3″), 111.9 C (C-3′), 108.8 CH (C-16), 40.1 CH_2_ (C-9), 29.5 CH_2_ (C-8), 23.4 CH_3_ (C-14/18).

### 4.5. Cytotoxicity Assays

Dragmacidin G (**1**) was evaluated for its effects on proliferation of the PANC-1 human pancreatic carcinoma (ATCC No. CRL-1469), MIA PaCa-2 human pancreatic carcinoma (ATCC No. CRL-1420), BxPC-3 human pancreatic adenocarcinoma (ATCC No. CRL-1687), and ASPC-1 human pancreatic adenocarcinoma (ATCC No. CRL-1682). The PANC-1, MIA PaCA-2, BxPC-3, and ASPC-1 cell lines were obtained from the American Type Culture Collection (ATCC; Rockville, MD, USA). Assays were run in 96-well plates. After 72 h of incubation with the compound, cytotoxicity was measured spectrophotometrically using MTT as the indicator. The details of the cytotoxicity assays have been described in an earlier publication [22]. All samples were assayed a minimum of three times to derive the final IC_50_ value. Results are presented as the average value ± standard deviation.

### 4.6. Antibiotic Assays

#### 4.6.1. *Staphylococcus aureus* and Methicillin-Resistant *Staphylococcus aureus*

MICs against *S. aureus* (ATCC #29213) and MRSA (ATCC #700787) were determined using a standard microdilution broth method with cation-supplemented Mueller-Hinton broth as the growth medium. All plates were incubated for 18 h at 37 °C. The MIC for the chloramphenicol positive control was 6.2 μg/mL against *S. aureus* and 3.1 μg/mL against MRSA. The MIC for the methicillin control was 3.1 μg/mL against *S. aureus* and >50 μg/mL against MRSA.

#### 4.6.2. *M. tuberculosis* Bioluminescent Growth Inhibition Assay

Antimycobacterial activity of the compound was tested using *M. tuberculosis* CDC1551 carrying the pMV306hsp-LuxG13 integrative plasmid, which provides constitutive expression of the *luxCDABE* operon. Cultures of the *Mtb*:*lux* strain at mid-log phase were diluted in 7H9 OADC to a final optical density (600 nm) of 0.01, and 24 μL of this inoculum was added in each well of 384 well plates. Dragmacidin G was dissolved in 100% dimethyl sulfoxide (DMSO) at 10 mg/mL and diluted in water to obtain a 5× working stock (1 mg/mL in 10% DMSO). Sixteen-point 2-fold serial dilutions of the compound were prepared in sterile medium at 5-fold the final concentration. Six microliters of the serial dilution were added to plates containing 24 μL of bacterial inoculum per well for a final volume of 30 μL per well. Final concentrations of compounds ranged from 200 μg/mL to 0.09 μg/mL, with each one tested in technical triplicates in two independent experiments. After 5 days of incubation at 37 °C, luminescence was measured using a Synergy H4 microplate reader (BioTek, Winooski, VT, USA). Each 384-well plate contained 8 replicate wells of the following controls: 2% DMSO and 10 μg/mL rifampicin.

### 4.7. Resazurin-Based Cytotoxicity Assay against J774 Macrophage Cells

J774 macrophages were cultured in Dulbecco’s minimal essential medium (DMEM) supplemented with 10% heat-inactivated fetal bovine serum. Cells (2.5 × 10^4^/well) were seeded in black clear-bottom 384-well plates the day before adding compounds and controls (2% DMSO and 2% Triton X-100 [final concentrations]). Cell survival was determined after 24 h by resazurin reduction measurement. Addition of 0.02 mg/mL resazurin was followed by 4 h of incubation at 37 °C and subsequent fluorescence reading (560 nm/590 nm) using a Synergy H4 plate reader (BioTek, Winooski, VT, USA). Data are presented as percent viability compared to the vehicle (2% DMSO) control, with 100% being noncytotoxic and 2% Triton X-100 control used to define 0% viable.

### 4.8. Anti-Plasmodial Assay

The IC_50_ value in the multi-drug resistant DD2 strain of the malarial parasite was determined using the SYBR Green-1 assay as described previously [5]. Selectivity of this class of inhibitor against malaria was determined by measuring cytotoxicity against NIH 3T3 fibroblasts using an MTS assay ((3-(4,5-dimethylthiazol-2-yl)-5-(3-carboxymethoxyphenyl)-2-(4-sulfophenyl)-2H-tetrazolium), CellTiter 96^®^ aqueous non-radioactive cell proliferation assay, (Promega Life Sciences, Fitchburg, WI, USA)).

## Figures and Tables

**Table 1:** ^1^H, ^13^C and ^15^N NMR data for dragmacidin G (**1**) (DMSO-*d*_6_, ^1^H 600 MHz; ^13^C 150 MHz).

Atom	δ_C_, Multiplicity	δ_N_	δ_H_ (*J* in Hz)	COSY	^1^H-^13^C HMBC ^b^	^1^H-^15^N HMBC ^c^	NOESY
1		324 ^a^					
2	150.1, C						
3	143.3, C						
4		299 ^a^					
5	135.4, CH		8.98, s		C2 (w), C3, C6, C3″	N1, N4	H2″, H4″
6	145.9, C						
8	28.7, CH_2_		3.56, m		C2, C9	N10	
9	40.2, CH_2_		3.54, m	H10	C8, C11		
10		80	7.93, bt (4.8)	H9ab	C8, C9, C11	N12, N13	H9ab
11	156.8, C						
12, 13		72					
1′	-	136	11.82, bs	H2′	C2′, C3′, C3a′, C7a′		H2′, H7′
2′	127.9, CH		8.08, d (2.7)	H1′	C3, C3′, C3a′, C7a′	N1′	H1′
3′	111.8, C						
3a′	125.4, C						
4′	123.1, CH		8.10, d (8.9)	H5′	C6′, C7a′		H5′
5′	122.8, CH		7.25, dd (8.9 1.4)	H4′, H7′	C3a′, C7′		H4′
6′	114.83, C						
7′ ^d^	114.4, CH		7.70, d (1.4)	H5′	C3a′, C5′, C7a′	N1′	
7a′	137.1, C						
1″	-	138	11.95, bs	H2″	C2″, C3″, C3a″, C7a″		H2″, H7″
2″	127.7, CH		8.33, d (2.7)	H1″	C6, C3″, C3a″, C7a″	N1″	H1″
3″	112.6, C						
3a″	124.0, C						
4″	122.6, CH		8.22, d (8.9)	H5″	C3″, C6″, C7a″		H5, H5″, H8ab
5″	123.3, CH		7.31, dd (8.9, 1.4)	H4″, H7″	C3a″, C7″		H4″
6″	114.78, C ^a^						
7″ ^d^	114.74, CH ^a^		7.71, d (1.4)	H5″	C3a″, C5″, C7a″	N1″	
7a″	137.9, C						

^a^ Assignments may be interchanged; ^b^ ^1^H-^13^C HMBC correlations, optimized for 8 Hz, are from proton(s) to the listed carbon; ^c^ ^1^H-^15^N HMBC correlations, optimized for 6 Hz are from the proton to the listed nitrogen atom; ^d^ H-7′ and H-7″ have substantial overlap but could be assigned based upon the ^1^H-^15^N HMBC experiment. This then allowed for the assignment of the ^1^H-^13^C HMBC assignments for H-7′ and H-7″ using the band selective HMBC experiment.

## Supplementary Materials

The following are available online at www.mdpi.com/1660-3397/15/1/15/s1: Figure S1: Description of the biological material, Figure S2: HRMS Data for dragmacidin G (**1**) with isotope matching, Figure S3: ^1^H NMR spectrum of dragmacidin G, Figure S4: ^13^C NMR spectrum of dragmacidin G, Figures S5 and S6: edited *g*-HSQC spectrum of dragmacidin G, Figures S7–S11: ^1^H-^13^C HMBC Spectrum of dragmacidin G, Figures S12–S16: Band Selective ^1^H-^13^C HMBC Spectrum of dragmacidin G, Figure S17: ^1^H-^15^N HMBC Spectrum of dragmacidin G, Figures S18–S21: ^1^H-^1^H 2D *g*-COSY spectrum of dragmacidin G, Figure S22: Measurement of ^1^J_CH_ for H-5 from residual one bond couplings observed in the HMBC spectrum of dragmacidin G, Figures S23 and S24: 2D-NOESY Spectrum of dragmacidin G, Figure S25: NMR data that support the presence of two 6-bromo-indol-3-yl rings in dragmacidin G, Figure S26: Analysis of Nuclear Overhouser Data from the 2D-*g*-NOESY spectrum, Figure S27: Pyrazine and Pyrazinine structures consistent with the HMBC data, Figure S28: Calculated NMR data for the pyrazine versus pyrazinine structure.
