# Supplementary material for: Dragmacidin G, a Bioactive Bis-Indole Alkaloid from a Deep-Water Sponge of the Genus Spongosorites
Source: Mar Drugs. 2017 Jan 11;15(1):16. doi: 10.3390/md15010016 (PMC5295236; doi:10.3390/md15010016)
Supplement: Supplementary file 1 [file marinedrugs-15-00016-s001.docx]

Supplementary Materials: Dragmacidin G,
a Bioactive Bis-Indole Alkaloid from a Deep-Water Sponge of the Genus *Spongosorites*

Amy E. Wright, K. Brian Killday, Debopam Chakrabarti, Esther Guzmán, Dedra Harmody,
Peter J. McCarthy, Tara Pitts, Shirley Pomponi, John K. Reed, Bracken F. Roberts,
Carolina Rodrigues Felix and Kyle H. Rohde

The sponge used in this study is an unidentified species of *Spongosorites* (Phylum Porifera, Class Demospongiae, Order Suberitida, Family Halichondriidae). It was collected from Long Island, Bahamas (23°41.12′ N, 75°22.18′ W), by the *Johnson-Sea-Link I* manned submersible at a depth of 630 m. It was thickly encrusting (approximately 5 cm thick × 20 cm long), firm in consistency, bright yellow alive, and dark brown in ethanol. Clusters of oscules are visible, scattered along the surface of the sponge. The ectosome is easily detachable. Spicules are oxeas, 400 μm long × 10 μm wide. Encrusting on approximately one-third of the surface is another sponge, white when live that is an unidentified species in the Class Demospongiae, Order Haplosclerida. This sponge is approximately 0.5 cm thick, brittle, with two size classes of oxeas (500 μm long × 15 μm wide, and 120 μm long × 7 μm wide). A taxonomic reference sample of both sponges is deposited in the Harbor Branch Oceanographic Museum, catalog number 003:00936 and is available for inspection upon request.


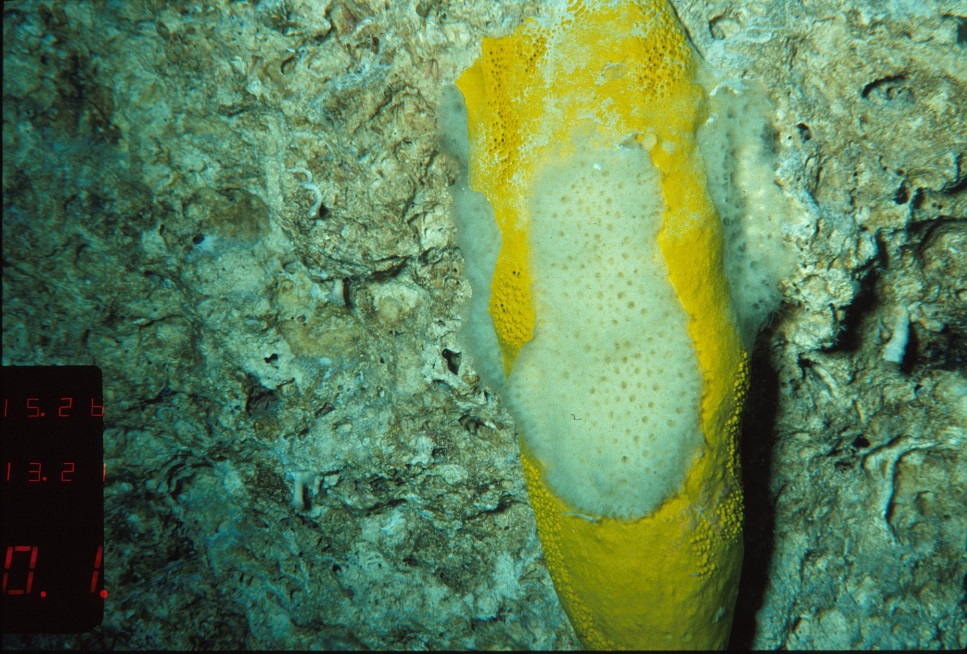


**Figure S1.** Description of Biological Material used in the study with in situ photograph.

|  |
| --- |
|  |

**Figure S2.** HRMS Data for dragmacidin G (**1**). (A) (i) HRMS of dragmacidin G was measured on a JEOL AccuTOF-DART 4G using a prototype paper spray attachment; (ii) 0.2 mg of dragmacidin G was dissolved in ~100 mL of methanol; (iii) 1–2 µL of dissolved sample were spotted onto the paper triangle (cut from Whatman No. 2 filter paper); (iv) The paper triangle was positioned to within ~5 mm of the apex of orifice 1 with the prototype paper spray accessory; (v) A potential of 2800 V was applied to the paper triangle and 20 µL of methanol were applied to the paper; (vi) The voltage for the atmospheric pressure interface are: Orifice 1 = 50 V; Orifice 2 = 5 V; Ring lens = 5 V; Ion guide voltage = 950 V; Orifice 1 temperature = 120 °C; (vii) Mass spectra were stored at a rate of 1 spectrum per second over the *m*/*z* range 100 to 800 and a resolving power of >10,000 (FWHM); (viii) Jeffamine M-600 was measured by DART as a reference standard within the same data file as the paper spray measurements. Dioctyl adipate peaks (present in the paper and detected as [M + Na]^+^ and [2M + Na]^+^) were used as internal drift correction peaks for maximum mass accuracy. (B) Isotope matching algorithms allow for the confirmation of sulfur in Dragmacidin G


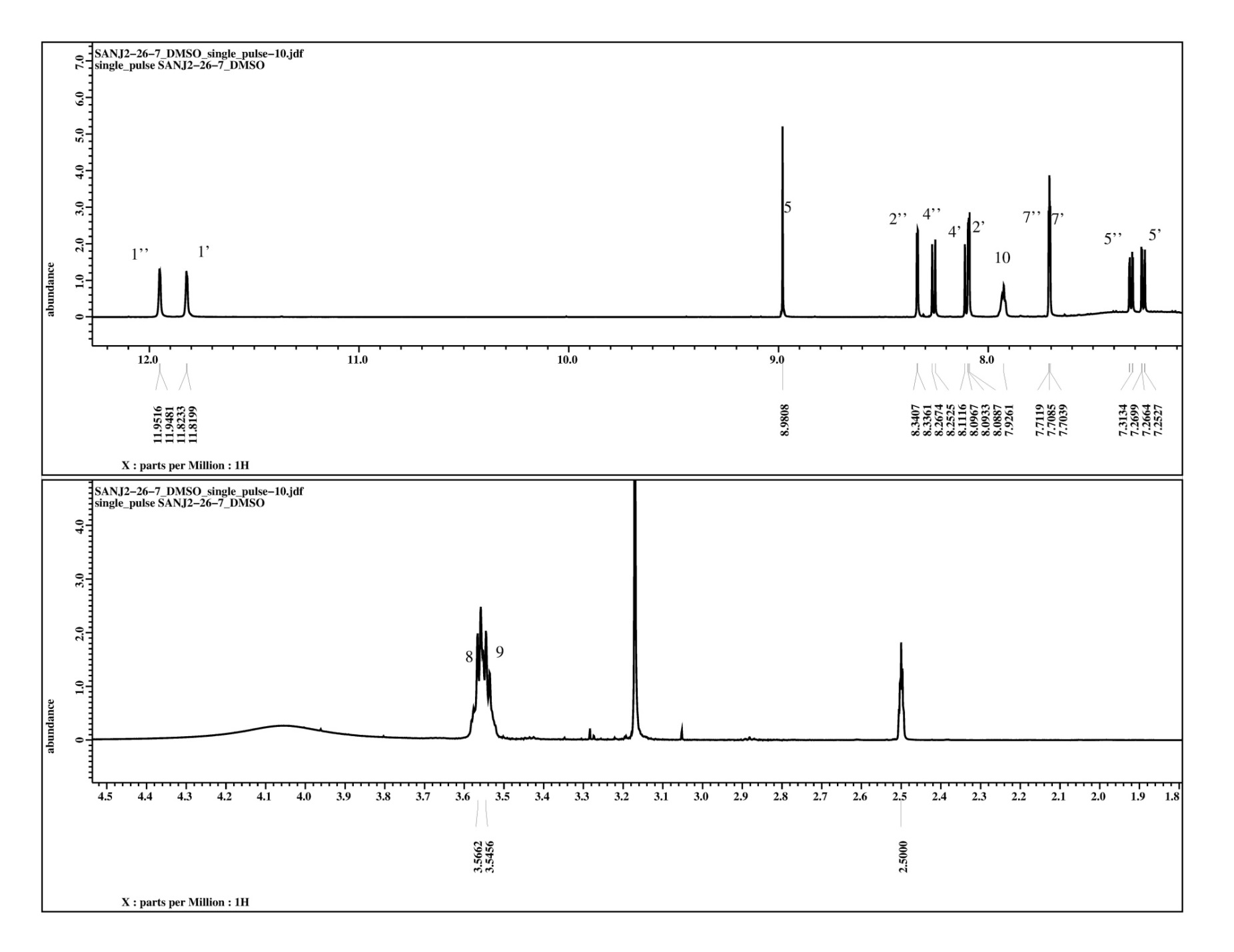


**Figure S3.** ^1^H NMR spectrum of dragmacidin G (600 MHz) DMSO-*d*_6_.


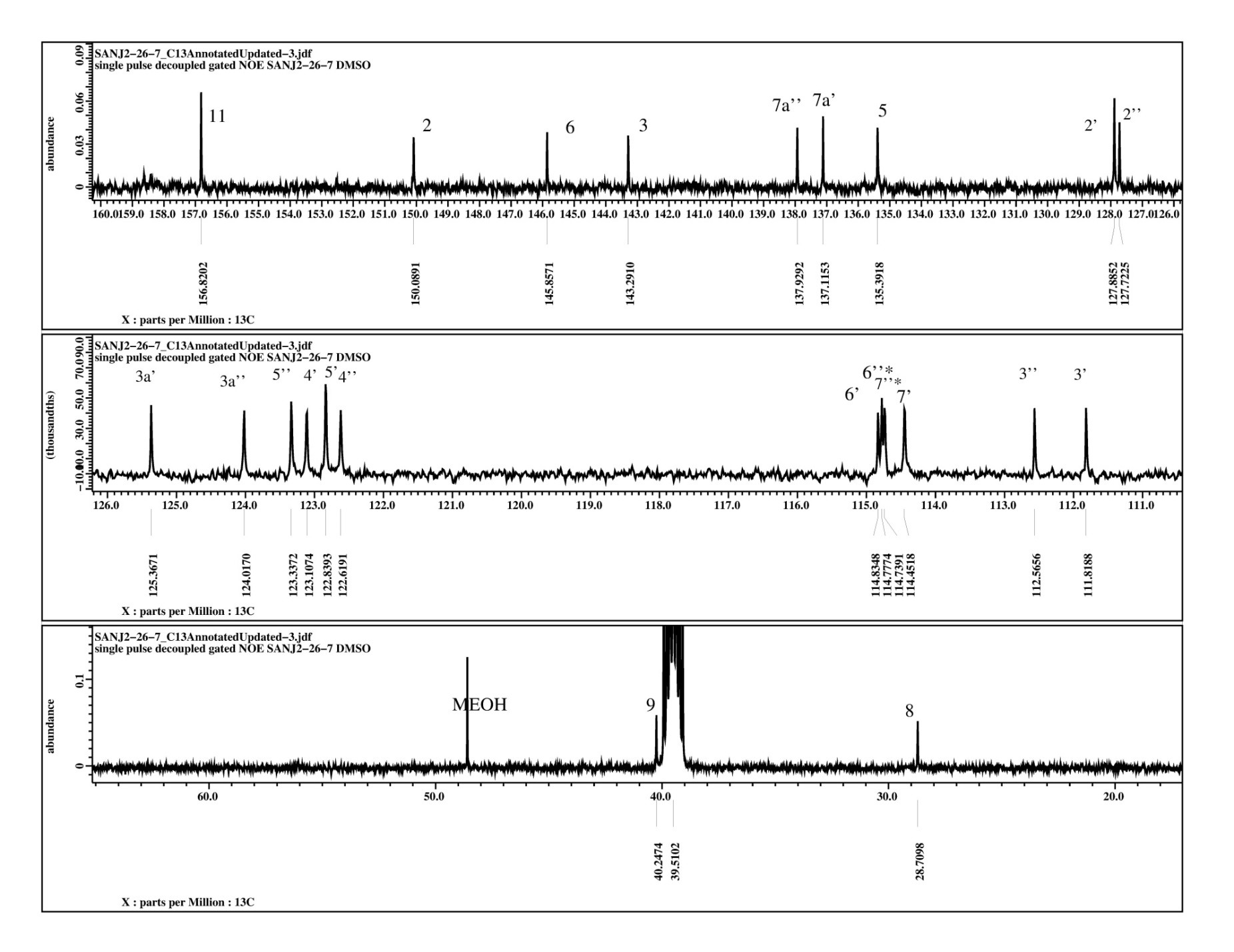


**Figure S4.** ^13^C NMR spectrum of dragmacidin G (150.1 MHz) DMSO-*d*_6_.


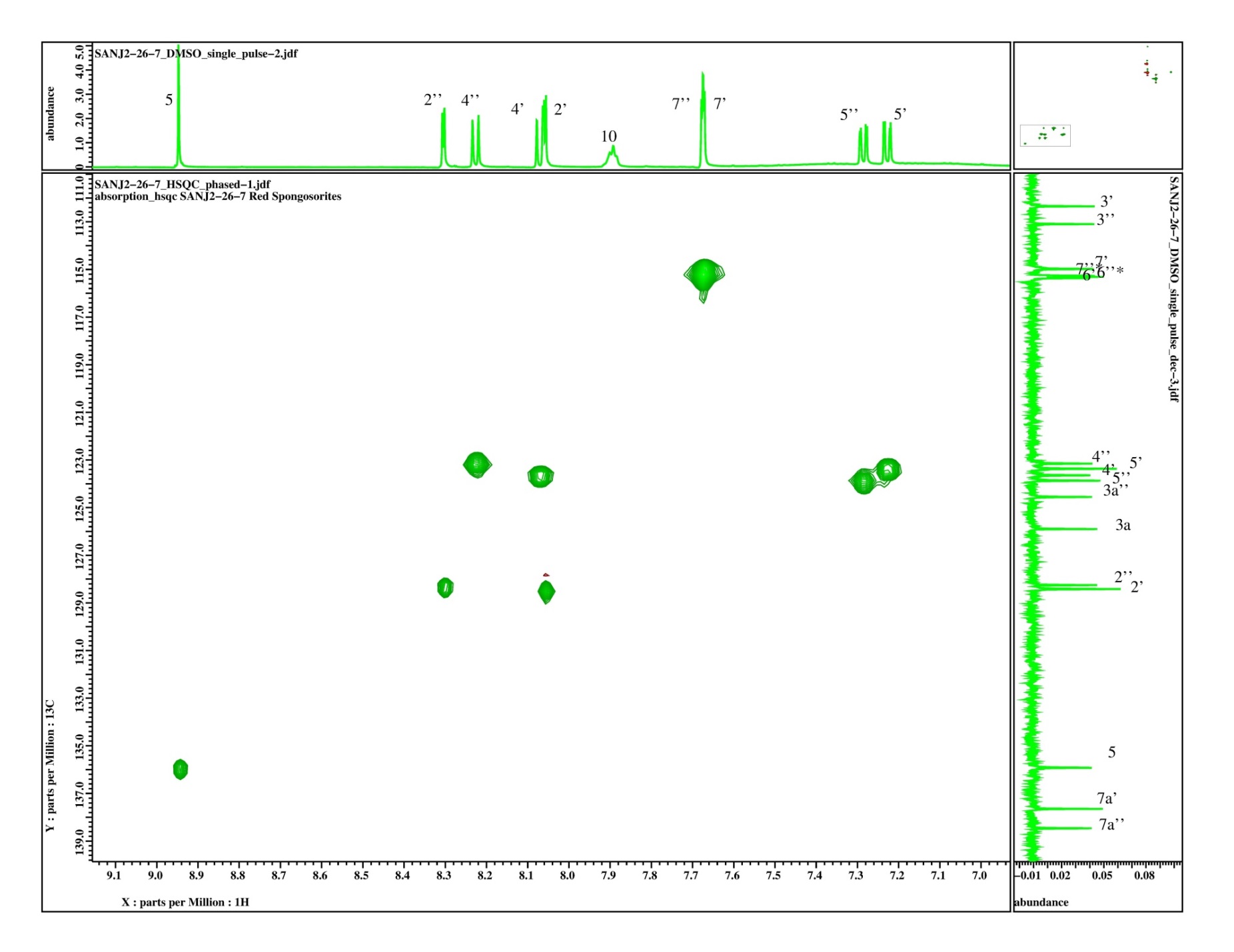


**Figure S5.** Expansion 1 of edited HSQC Spectrum of dragmacidin G (600 MHz) DMSO-*d*_6_.


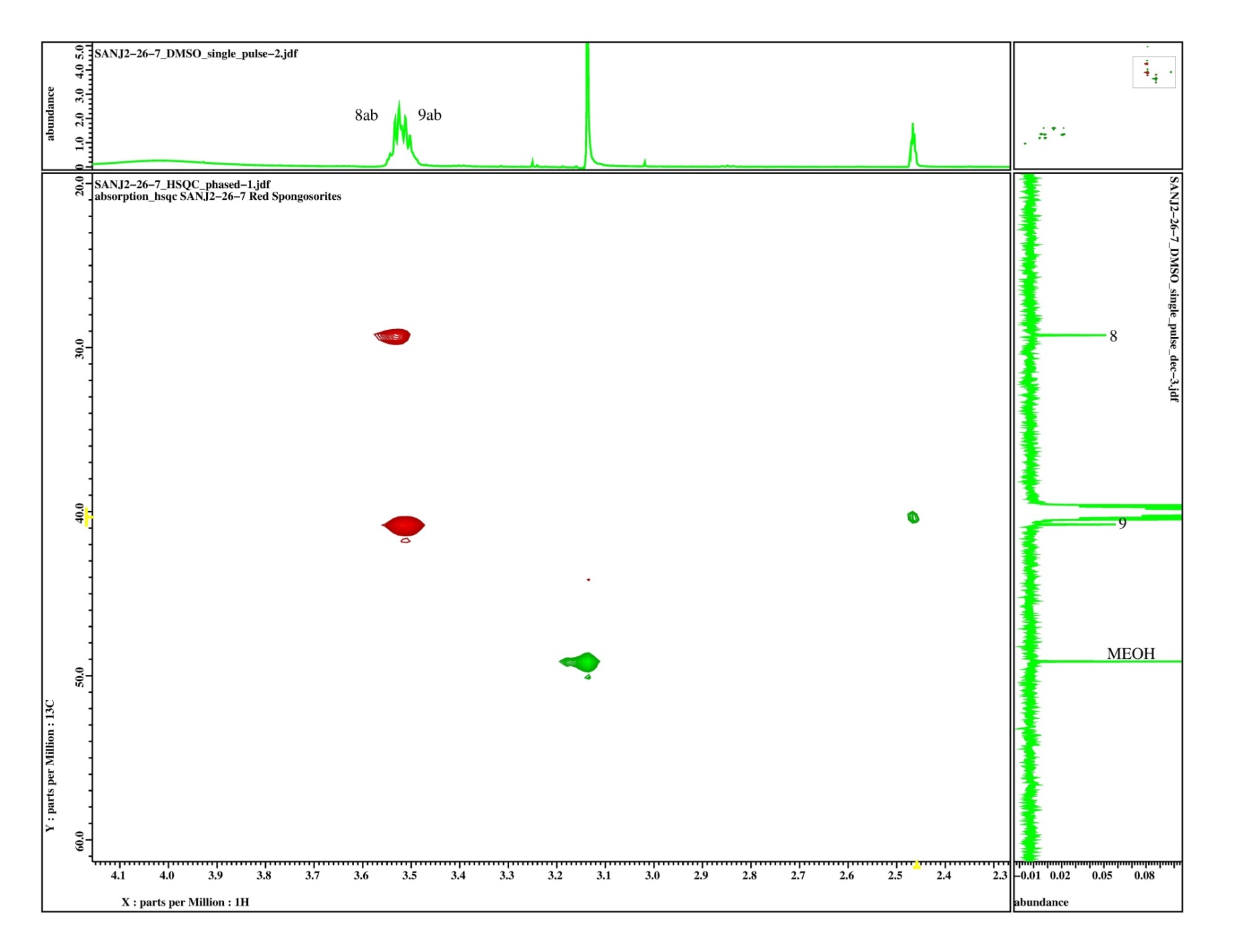


**Figure S6.** Expansion 2 of edited HSQC Spectrum of dragmacidin G (600 MHz) DMSO-*d*_6_.


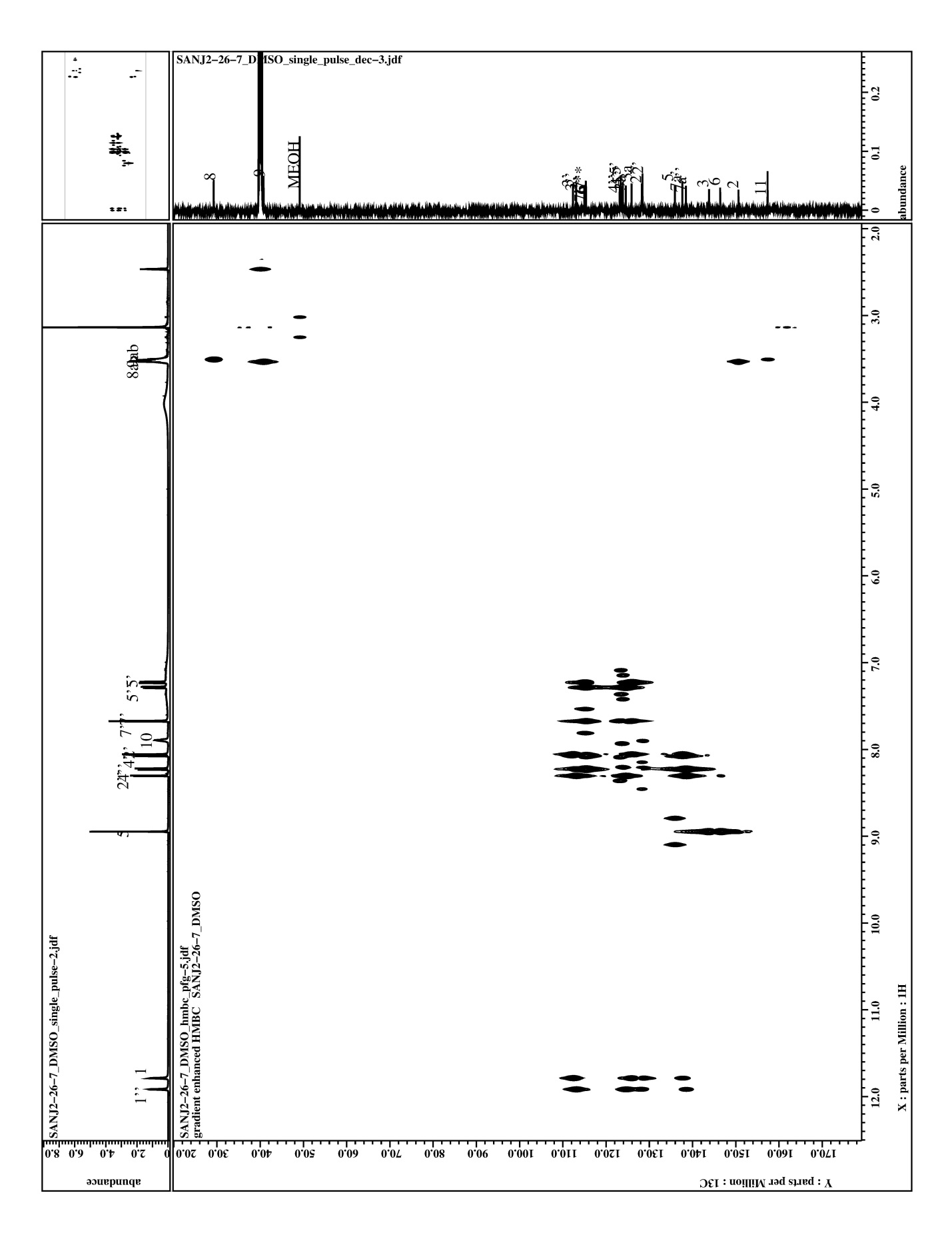


**Figure S7.** ^1^H-^13^C HMBC Spectrum of dragmacidin G (600 MHz) DMSO-*d*_6_.


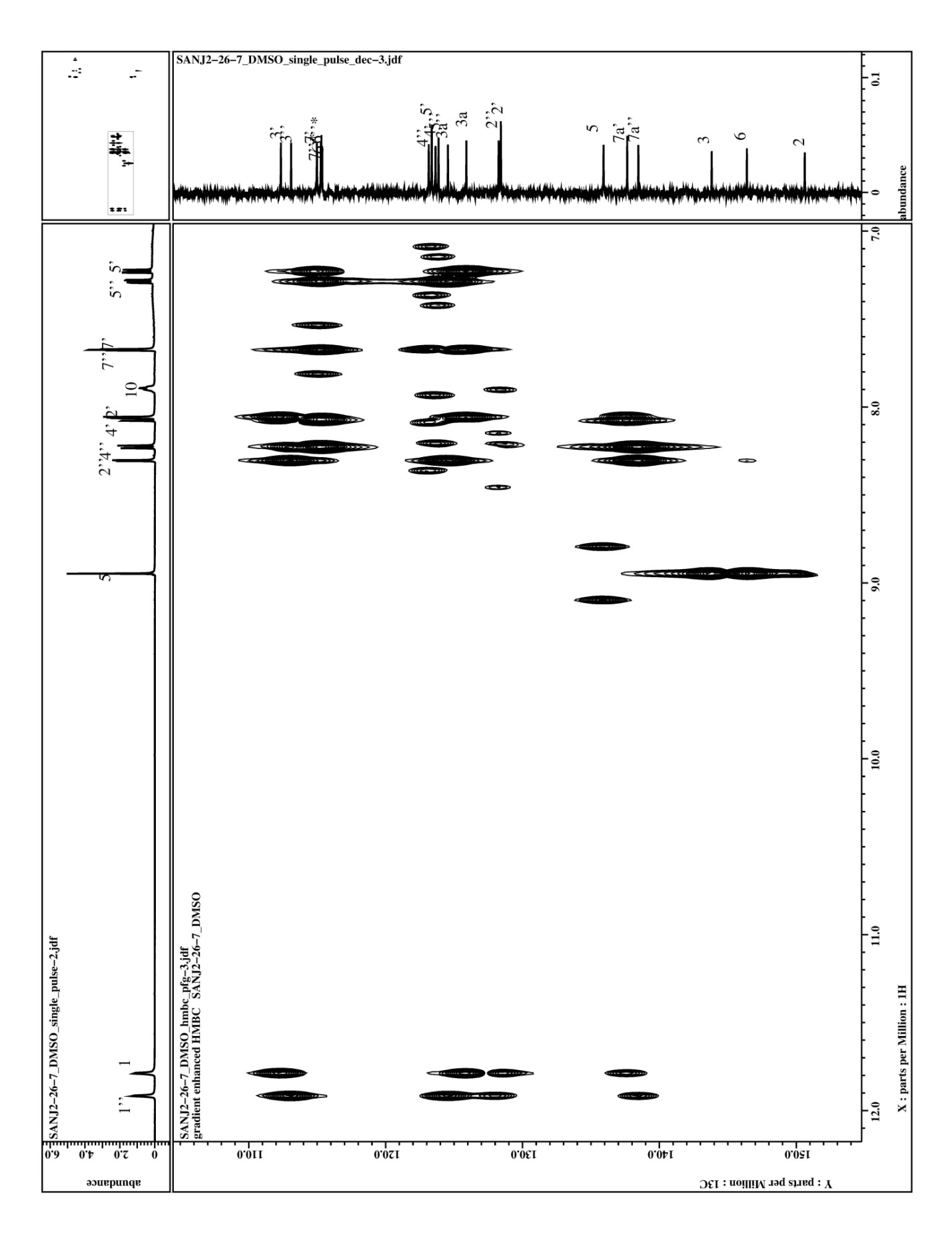


**Figure S8.** Expansion Number 1 of ^1^H-^13^C HMBC Spectrum of dragmacidin G (600 MHz) DMSO-*d*_6_.


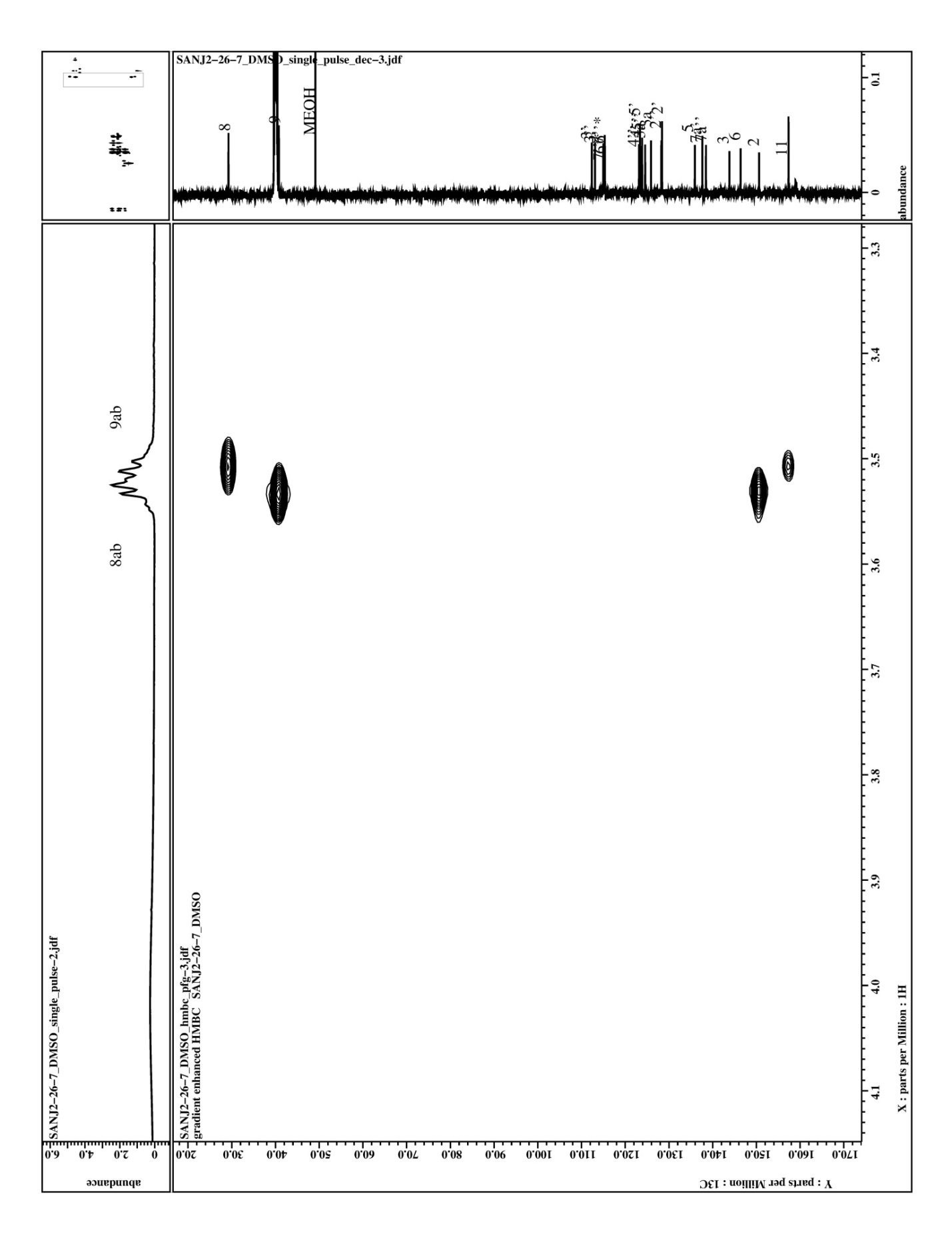


**Figure S9.** Expansion Number 2 of ^1^H-^13^C HMBC Spectrum of dragmacidin G (600 MHz) DMSO-*d*_6_.


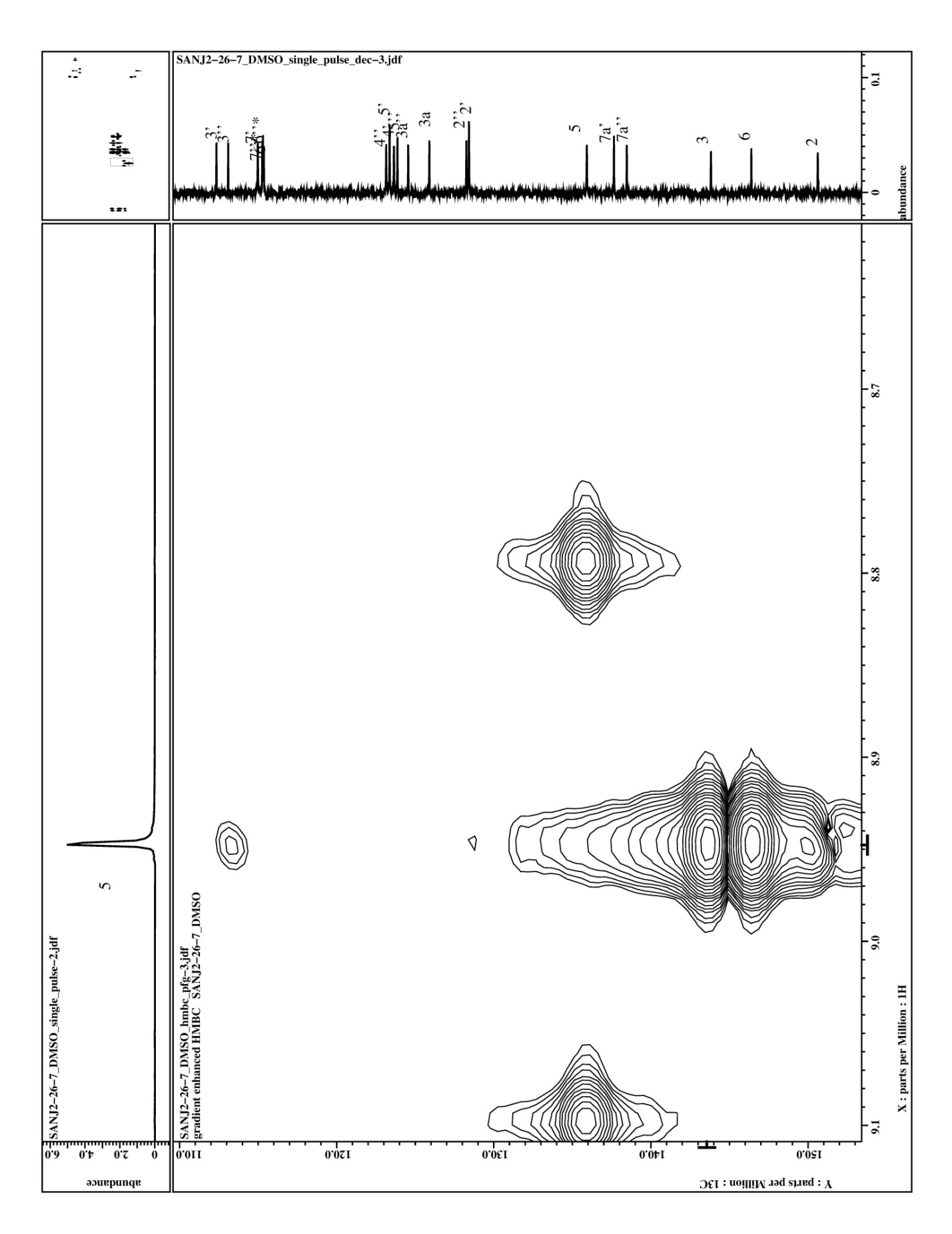


**Figure S10.** Expansion Number 3 of ^1^H-^13^C HMBC Spectrum of dragmacidin G (600 MHz) DMSO-*d*_6_.


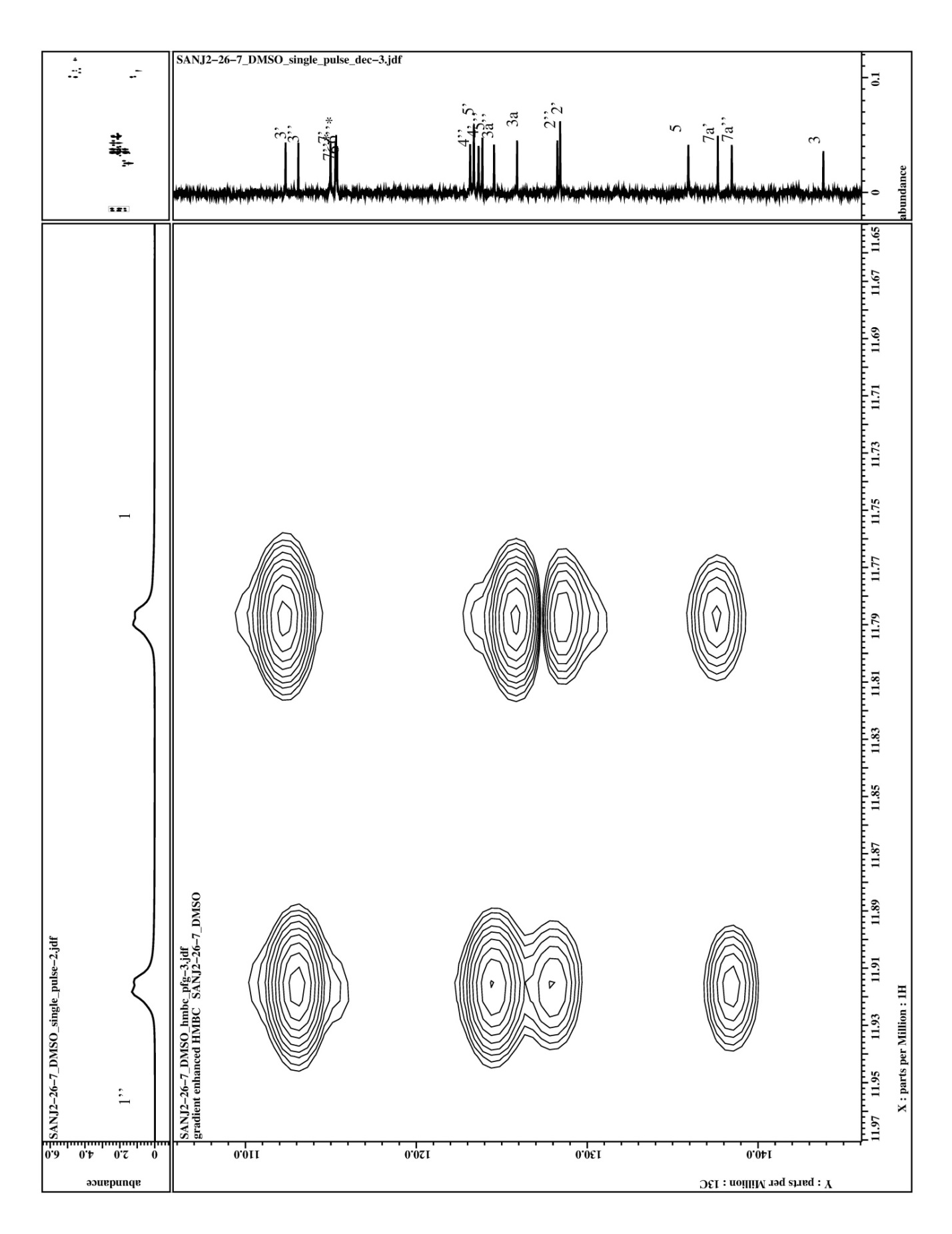


**Figure S11.** Expansion Number 4 of ^1^H-^13^C HMBC Spectrum of dragmacidin G (600 MHz) DMSO-*d*_6_.


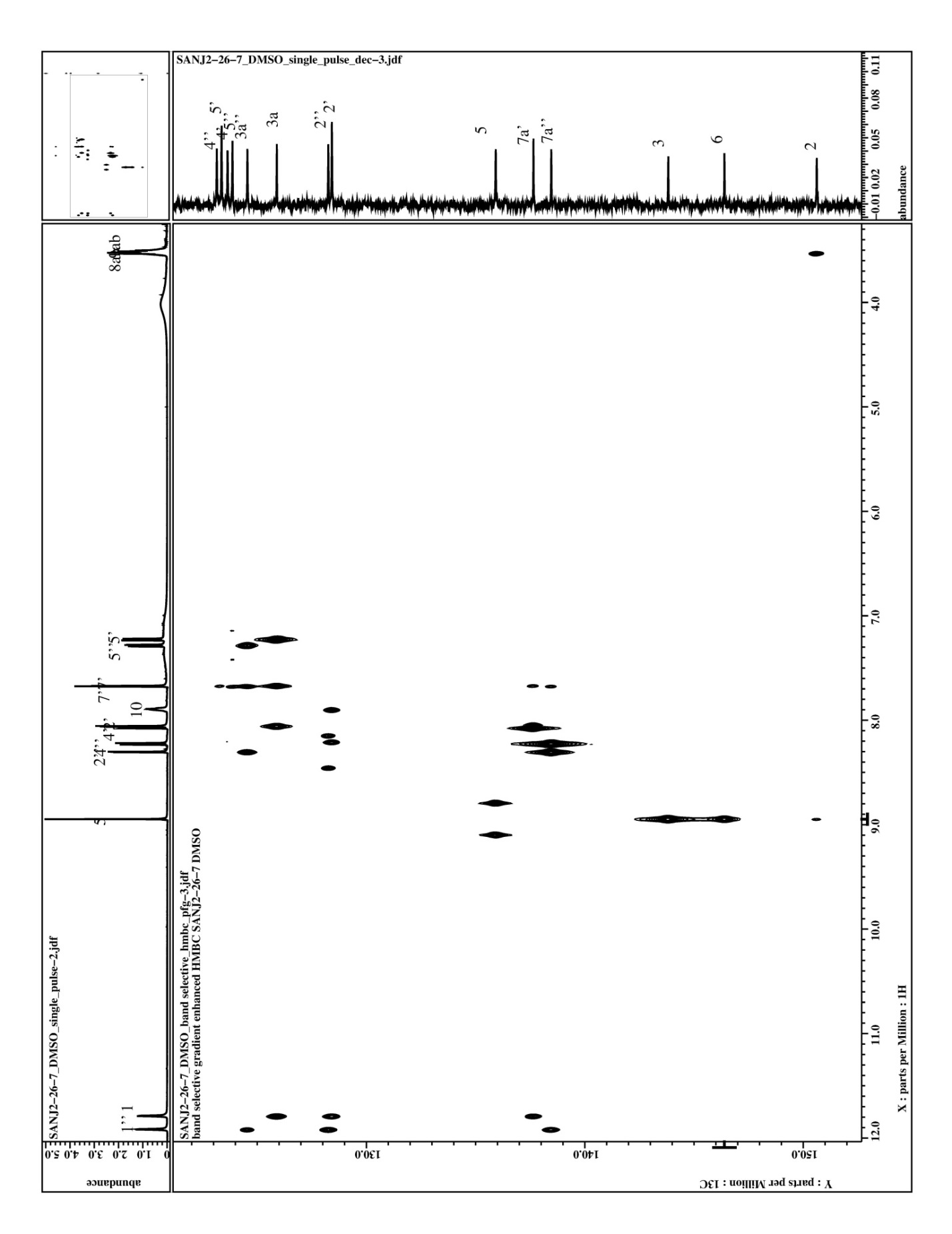


**Figure S12.** Band Selective ^1^H-^13^C HMBC Spectrum of dragmacidin G (600 MHz) DMSO-*d*_6_.


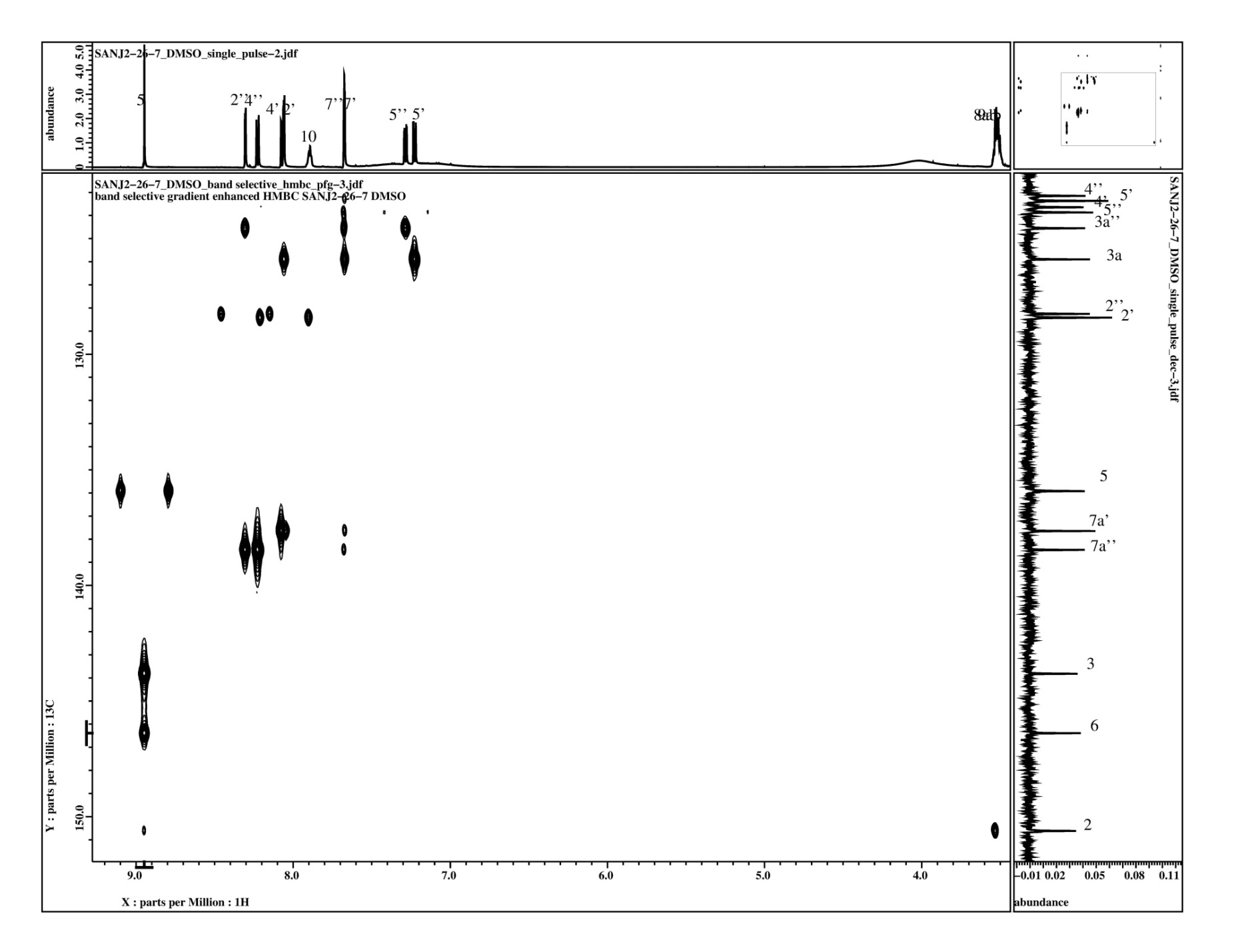


**Figure S13.** Expansion Number 1 of Band Selective ^1^H-^13^C HMBC Spectrum of dragmacidin G (600 MHz) DMSO-*d*_6_.


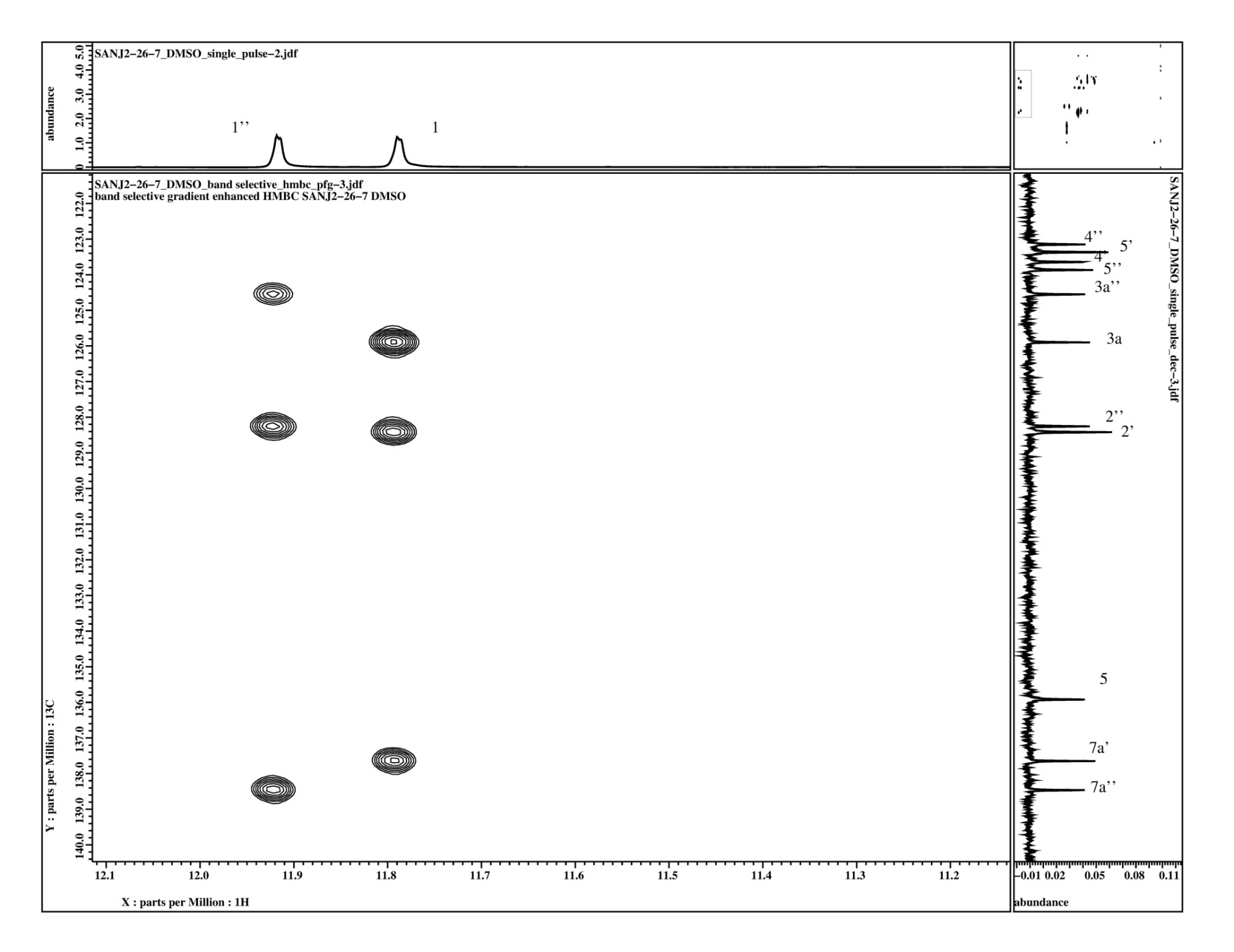


**Figure S14.** Expansion Number 2 of Band Selective ^1^H-^13^C HMBC Spectrum of dragmacidin G (600 MHz) DMSO-*d*_6_.


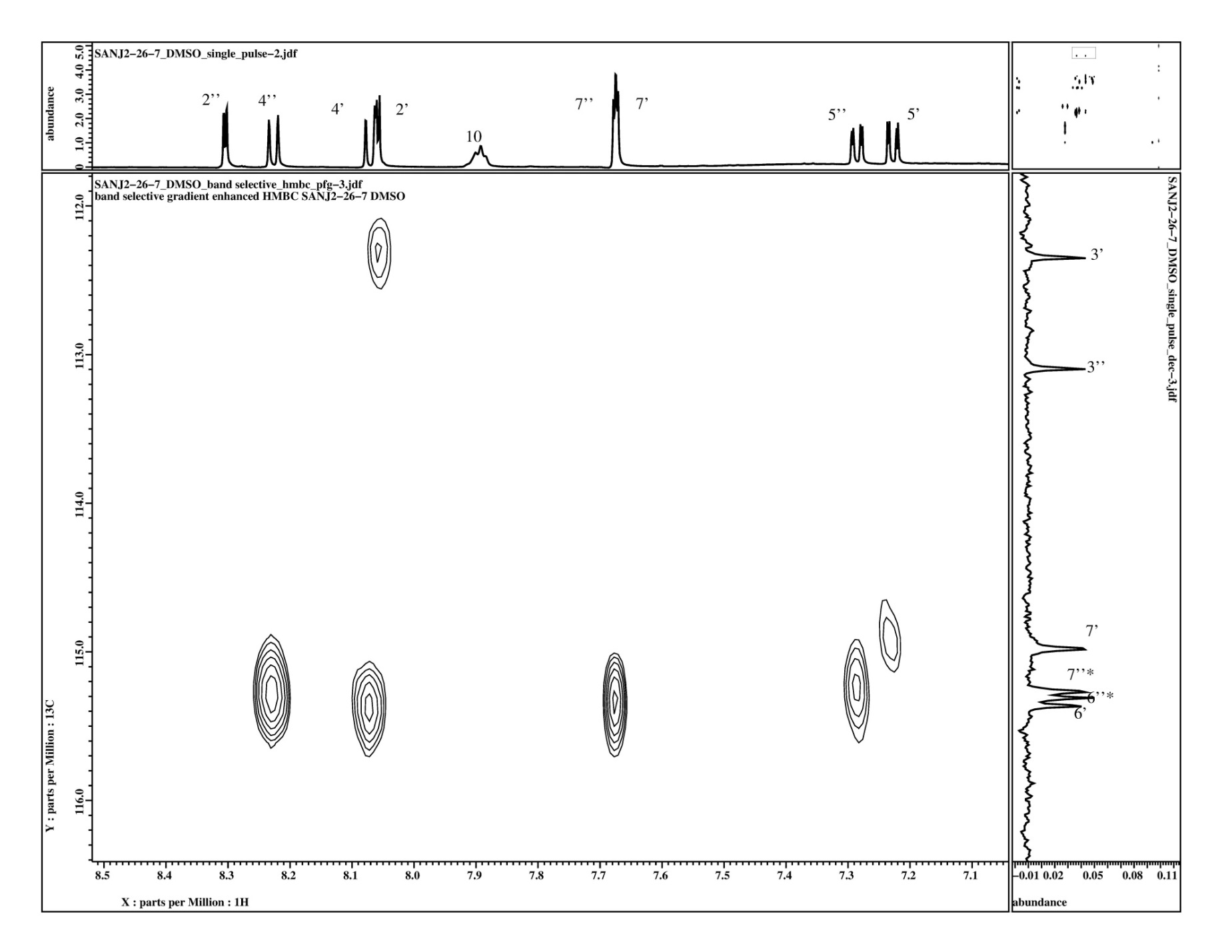


**Figure S15.** Expansion Number 3 of Band Selective ^1^H-^13^C HMBC Spectrum of dragmacidin G (600 MHz) DMSO-*d*_6_.


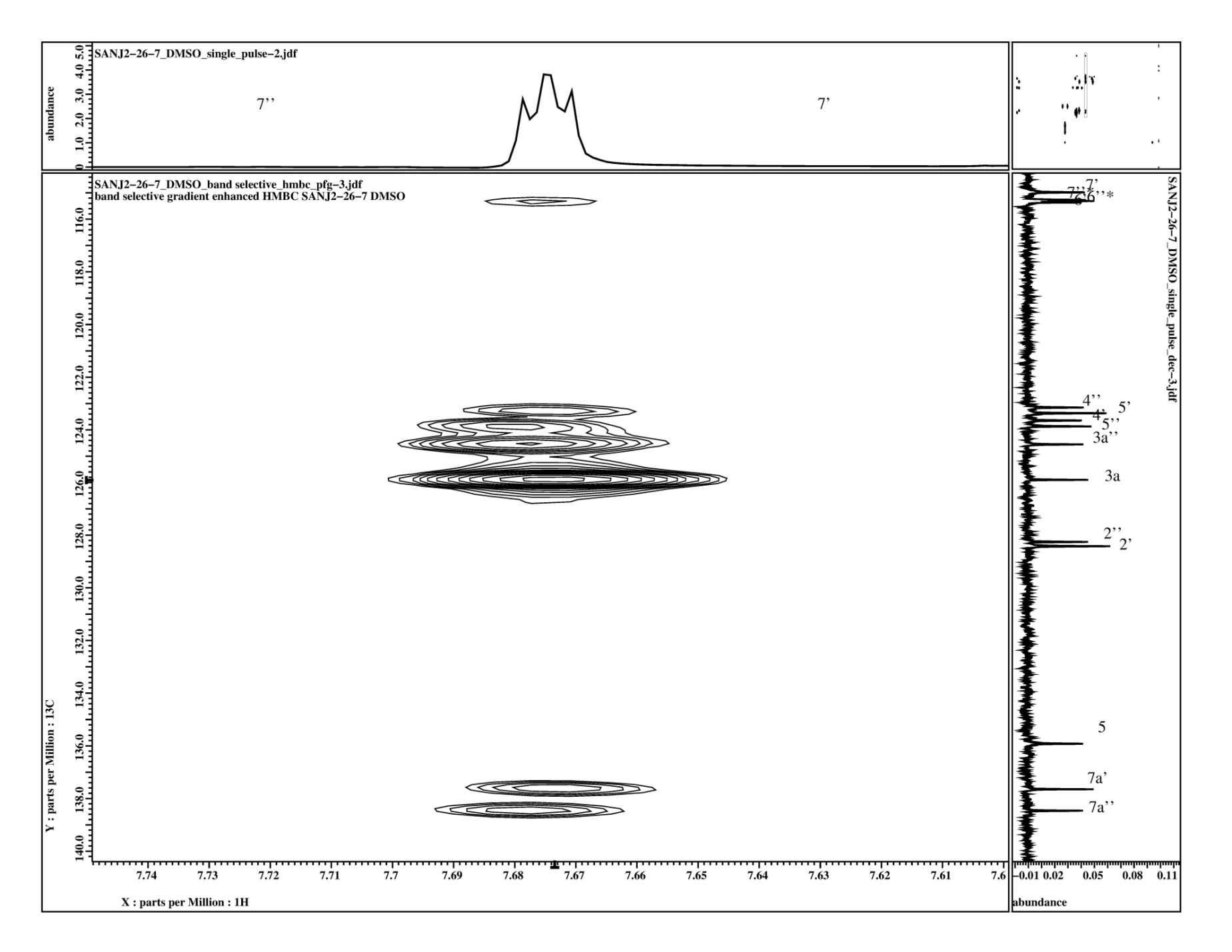


**Figure S16.** Expansion Number 4 of Band Selective ^1^H-^13^C HMBC Spectrum of dragmacidin G (600 MHz) DMSO-*d*_6_.


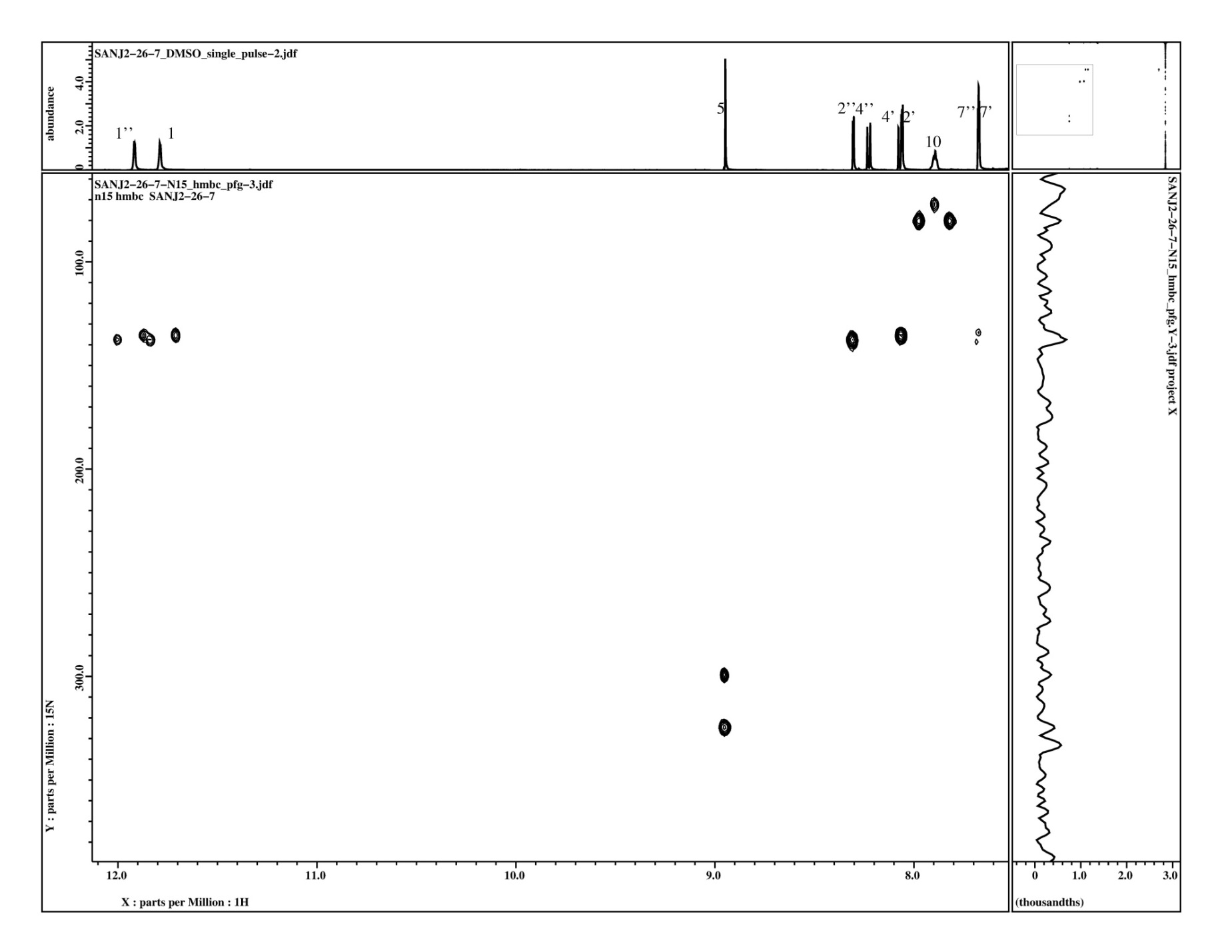


**Figure S17.** ^1^H-^15^N HMBC Spectrum of dragmacidin G (600 MHz) DMSO-*d*_6_.


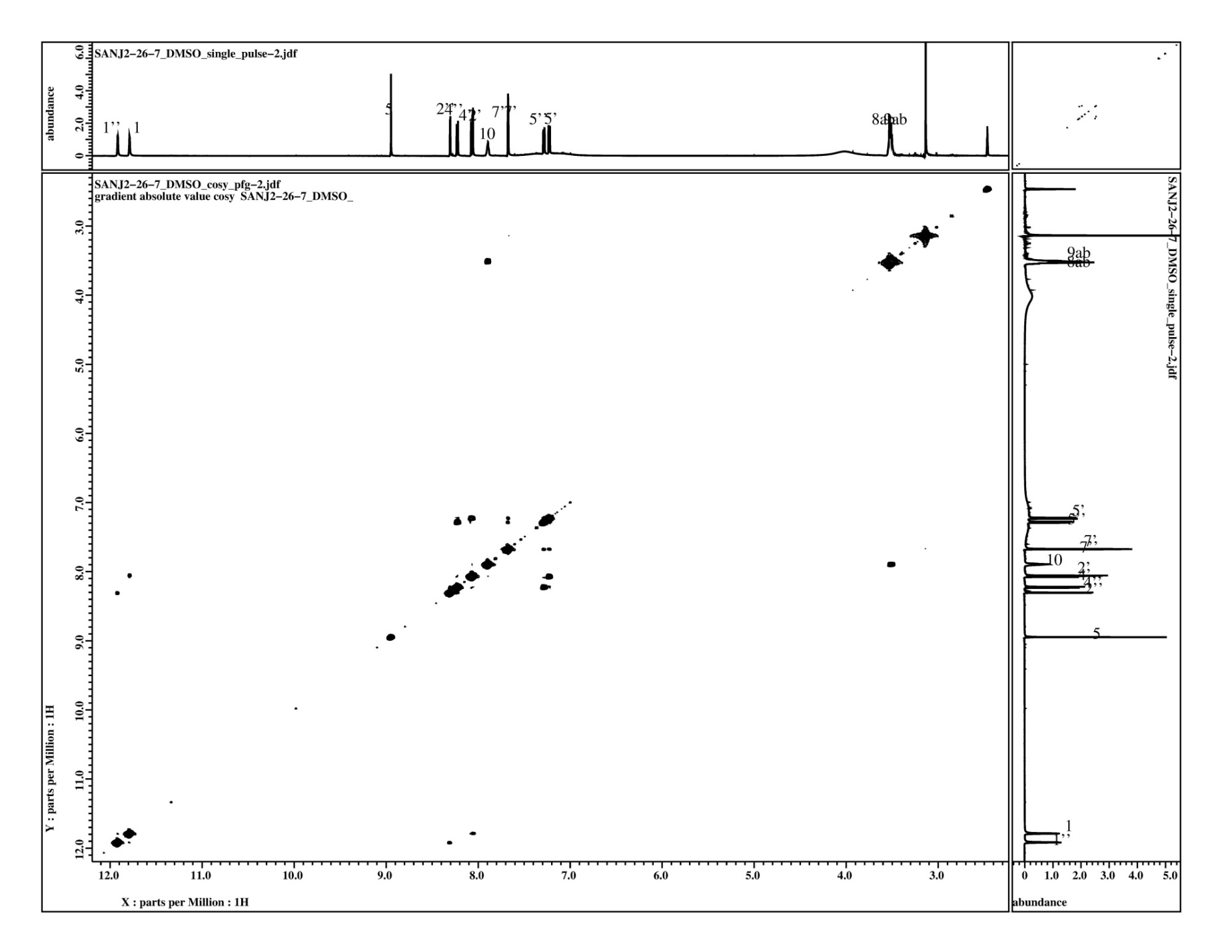


**Figure S18.** ^1^H-^1^H g-COSY spectrum of dragmacidin G (600 MHz) DMSO-*d*_6_.


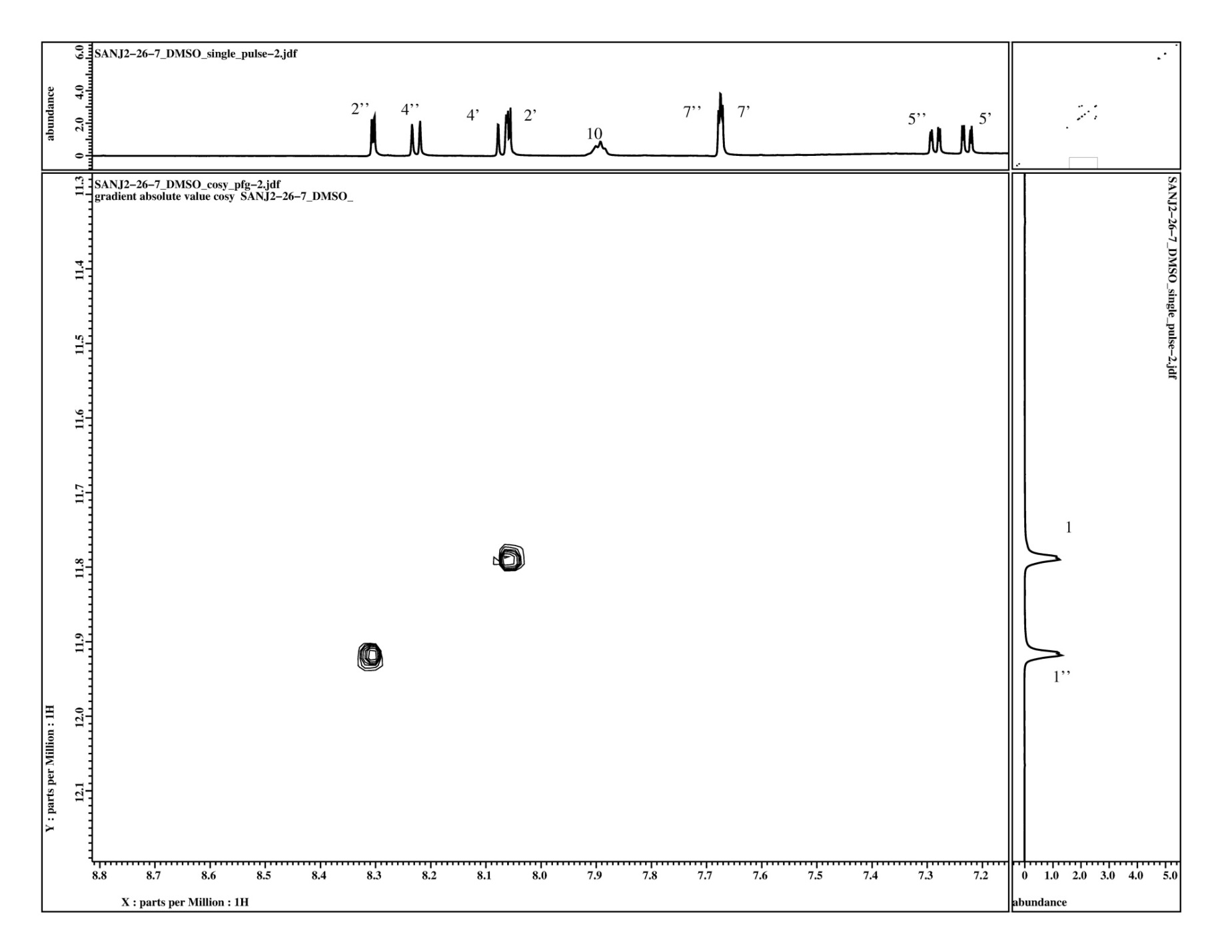


**Figure S19.** Expansion Number 1 of ^1^H-^1^H g-COSY spectrum of dragmacidin G (600 MHz) DMSO-*d*_6_.


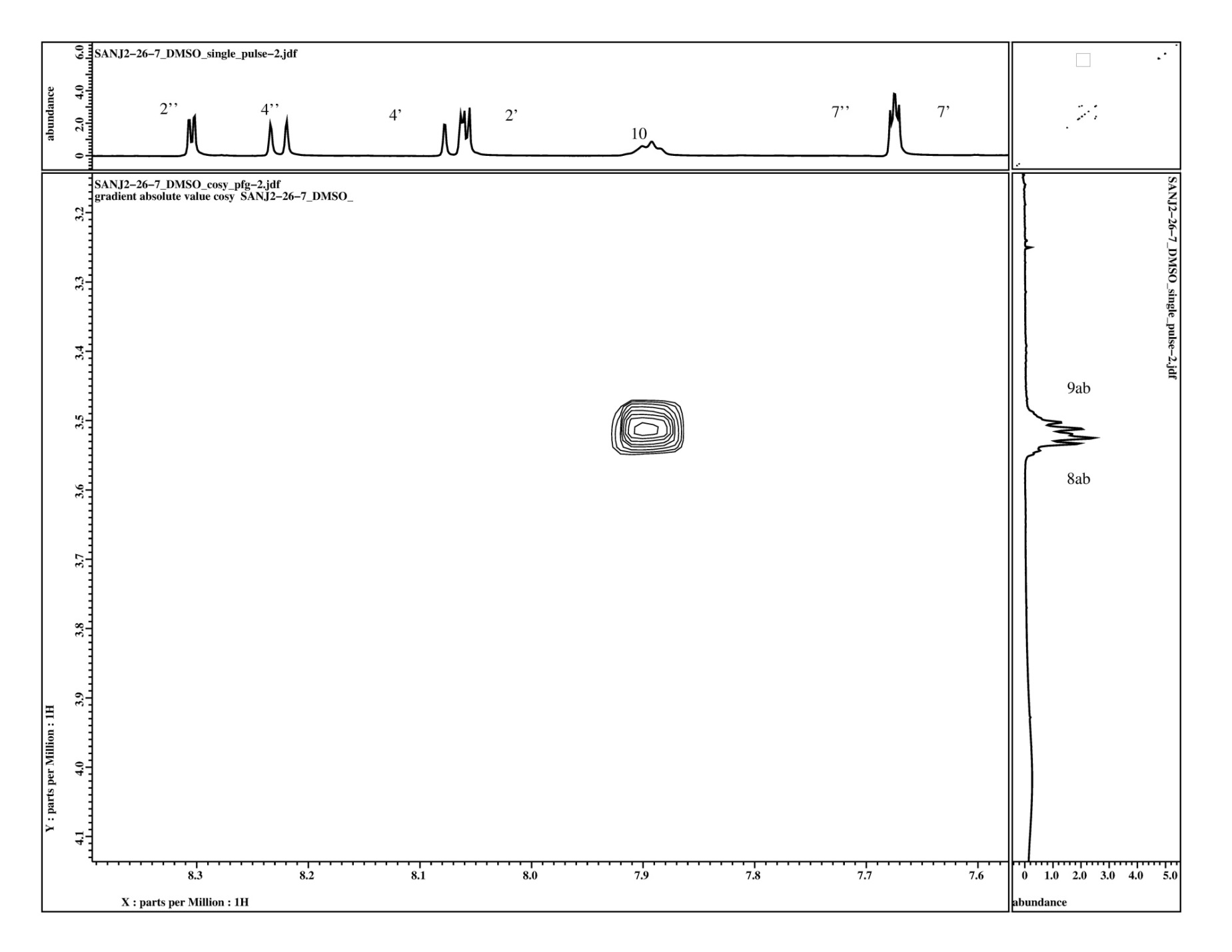


**Figure S20.** Expansion Number 2 of ^1^H-^1^H g-COSY spectrum of dragmacidin G (600 MHz) DMSO-*d*_6_.


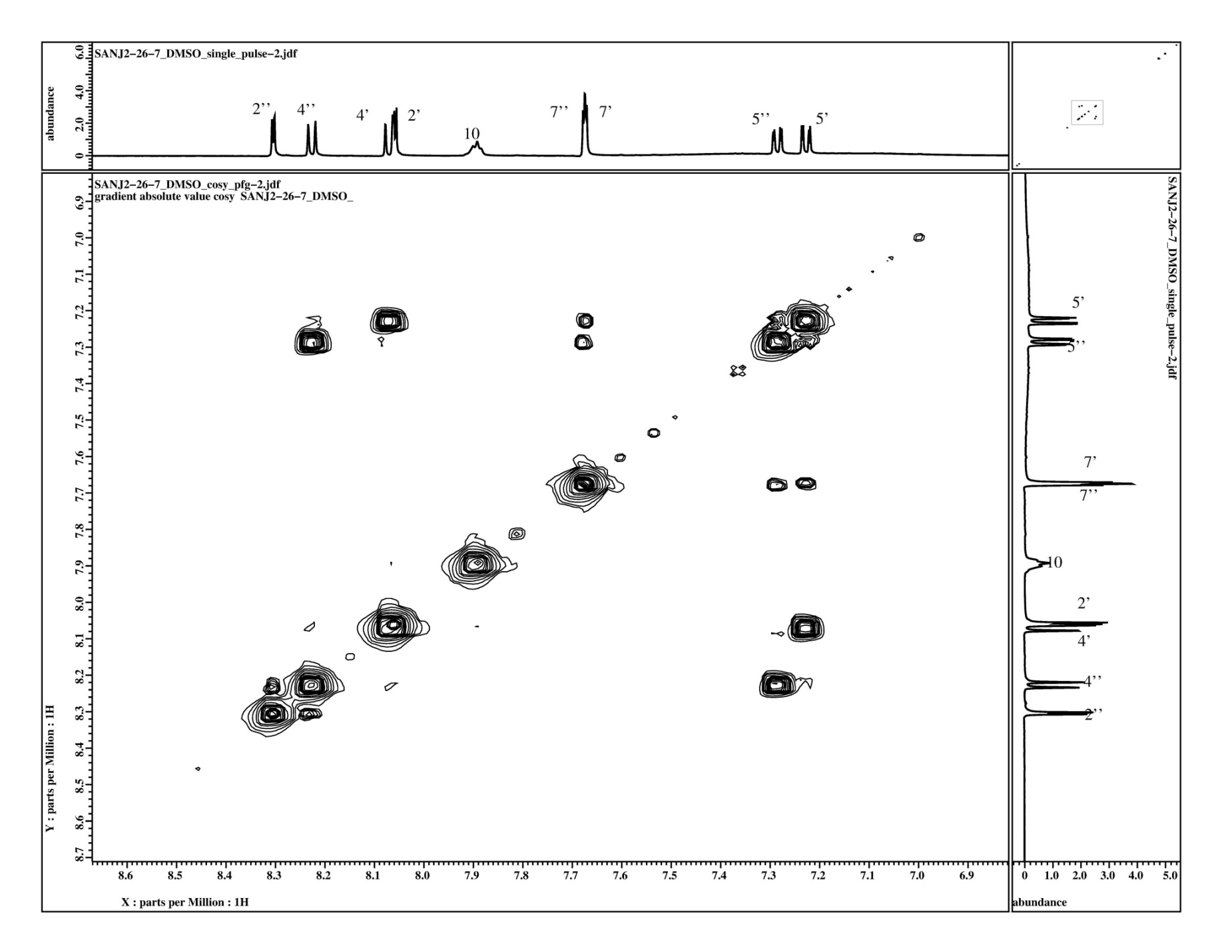


**Figure S21.** Expansion Number 3 of ^1^H-^1^H g-COSY spectrum of dragmacidin G (600 MHz) DMSO-*d*_6_.


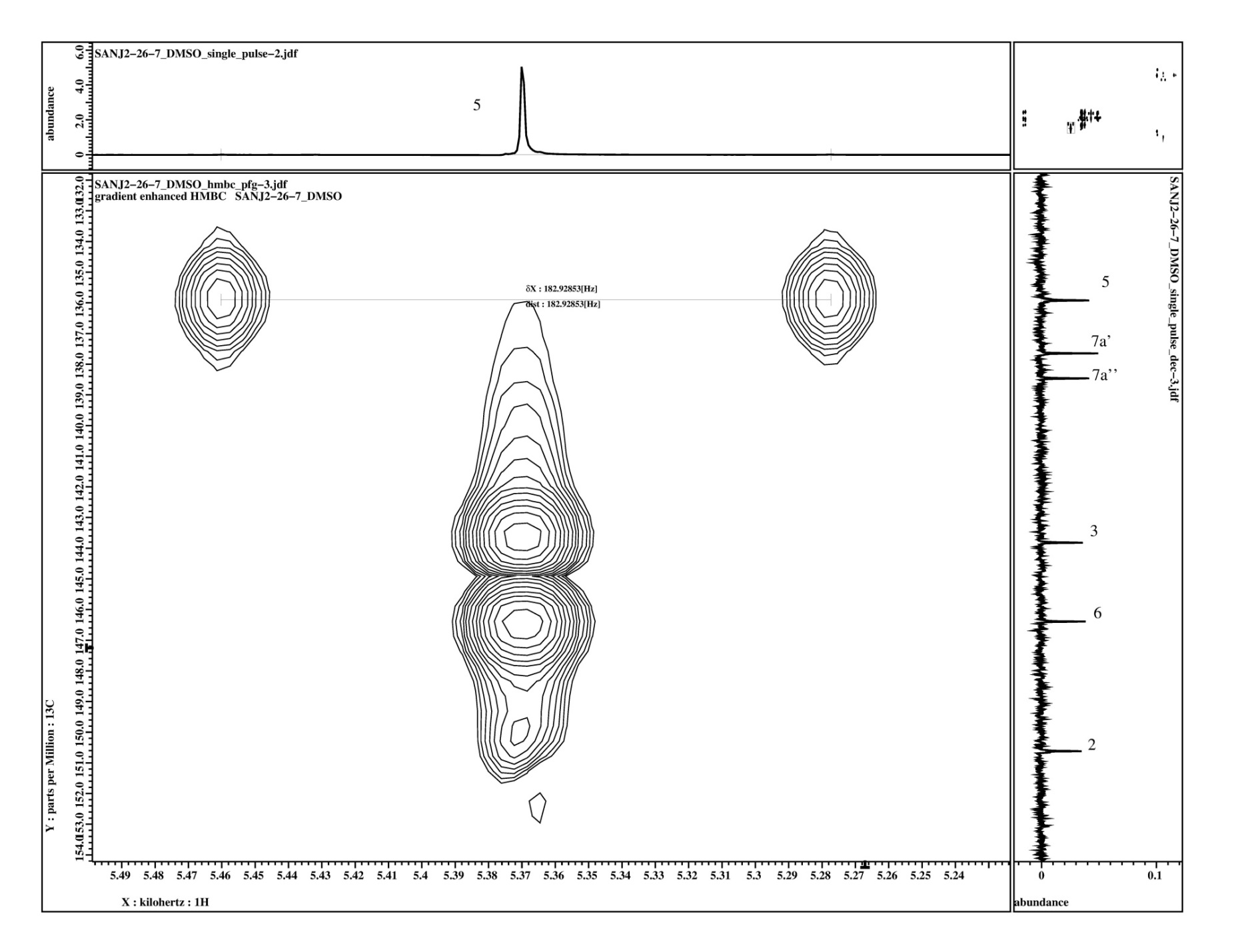


**Figure S22.** Measurement of ^1^J_CH_ for H-5 from residual one bond couplings observed in the HMBC spectrum of dragmacidin G (600 MHz) DMSO-*d*_6_.


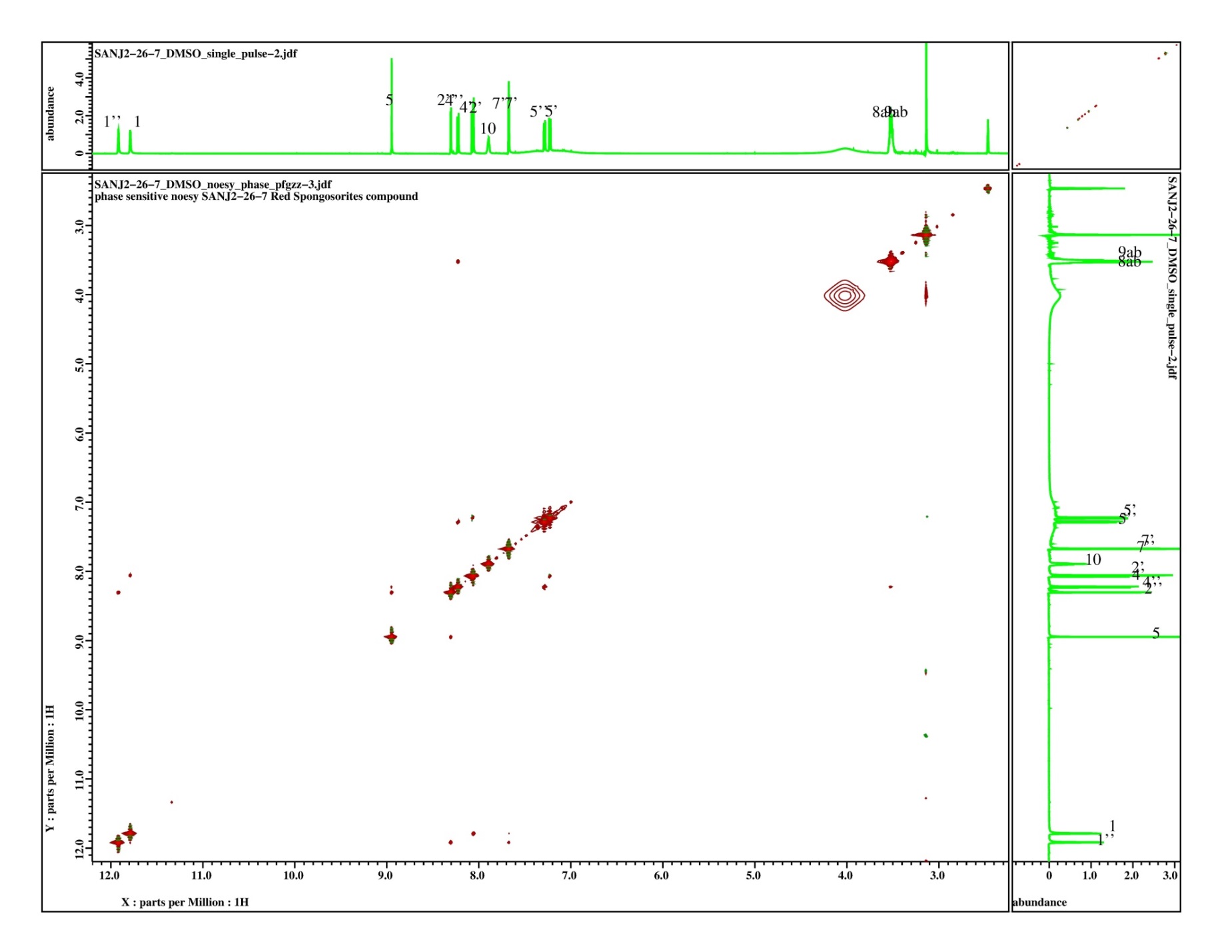


**Figure S23.** 2D-NOESY Spectrum of dragmacidin G (600 MHz) DMSO-*d*_6_.


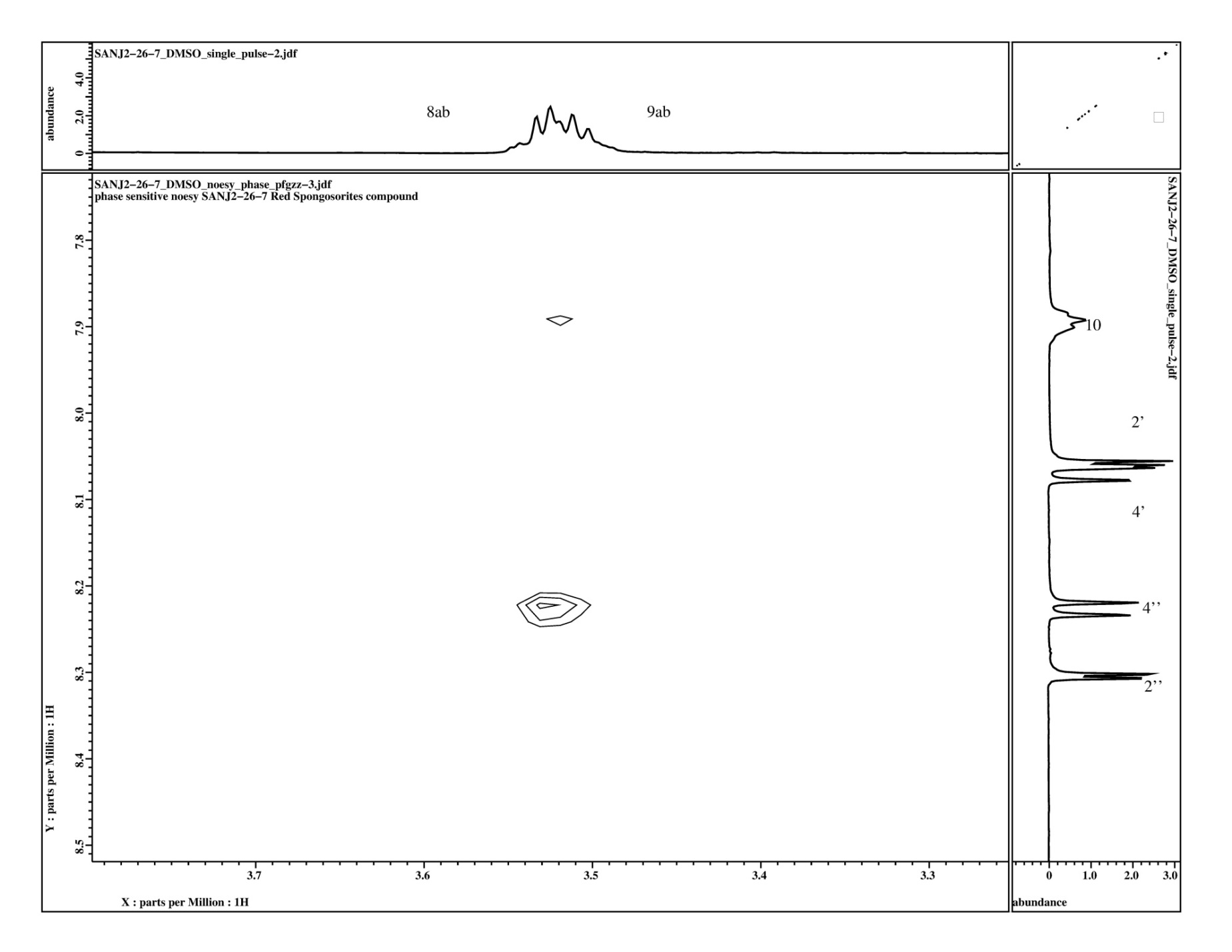


**Figure S24.** Expansion Number 1 of the 2D-NOESY Spectrum of dragmacidin G (600 MHz) DMSO-*d*_6_.

| HMBC and COSY data that support the “A” indole functionality |
| --- |
|  |
| HMBC and COSY data that support the “B” indole functionality |
|  |

**Figure S25.** NMR data that support the presence of two 6-bromo-indol-3-yl rings in dragmacidin G.

|  |
| --- |
| (**a**) |
|  |
| (**b**) |

**Figure S26.** Analysis of Nuclear Overhouser Data from the 2D-*g*-NOESY (600 MHz, DMSO-*d*_6_). (**a**) In the pyrazine structure correlations are observed in the 2D-NOESY spectrum between the H-5 methine proton and both H-2″ and H-4″ of the “B” ring indole. Strong correlations are also observed between H_2_-8 of the *N*-(2-mercaptoethyl) guanidine side chain and H-4″ of the B-ring indole. The near 90 degree angle of the CSC bond places these protons in close proximity; (**b**) in the pyrazinine structure (below), the two indole rings are on adjacent carbons creating a more sterically crowded molecule. One might expect some nOe between these protons. None are observed. In the pyrazinine structure the *N*-(2-mercaptoethyl) guanidine side chain is adjacent to the “A” ring indole. No correlations are observed between the side chain protons and the A ring indole protons.

|  |  |
| --- | --- |
| **1** | **3** |

**Figure S27.** Pyrazine (**1**) and Pyrazinine (**3**) structures consistent with HMBC data.

|  |
| --- |
| (**a**) |
| 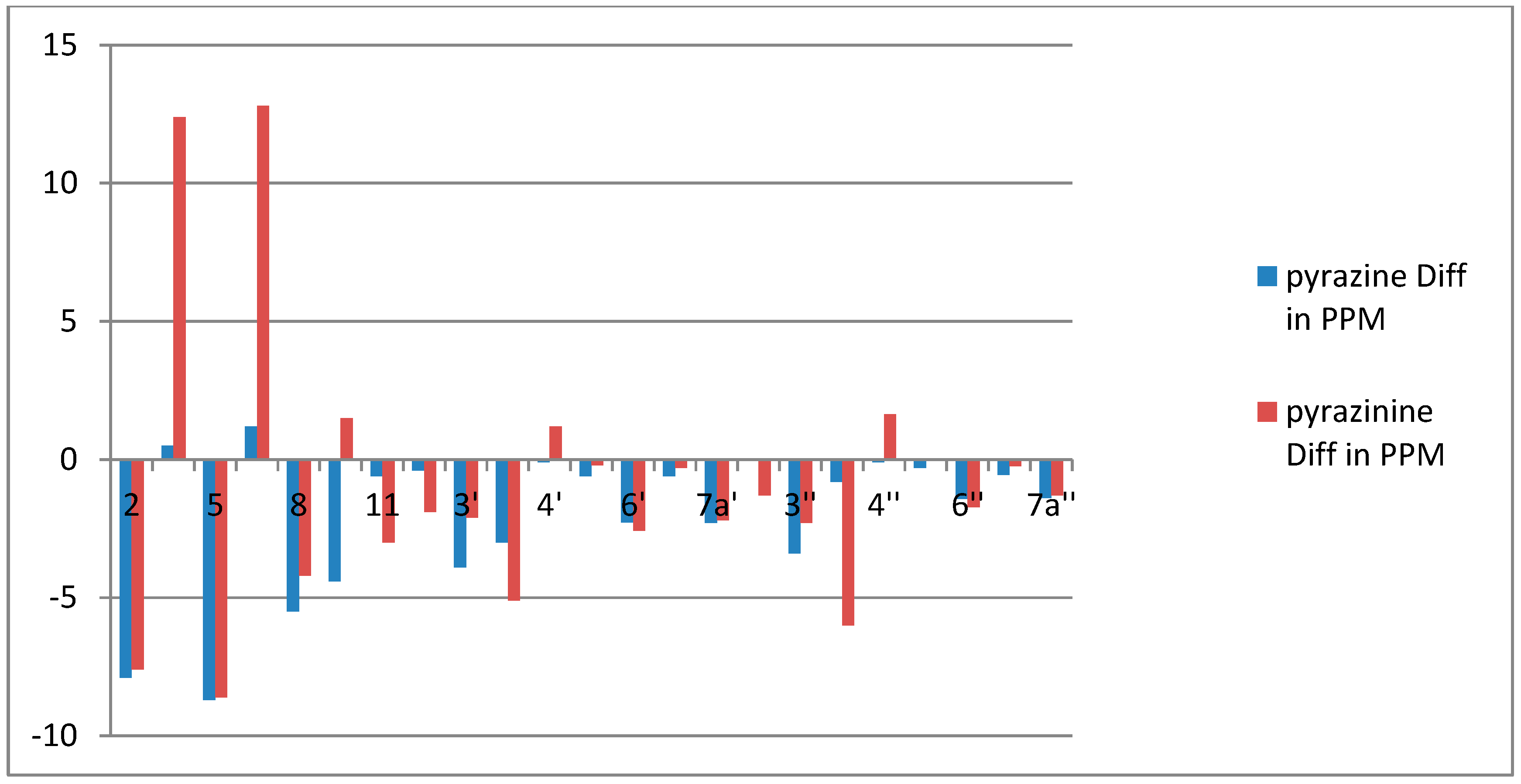 |
| (**b**) |
| 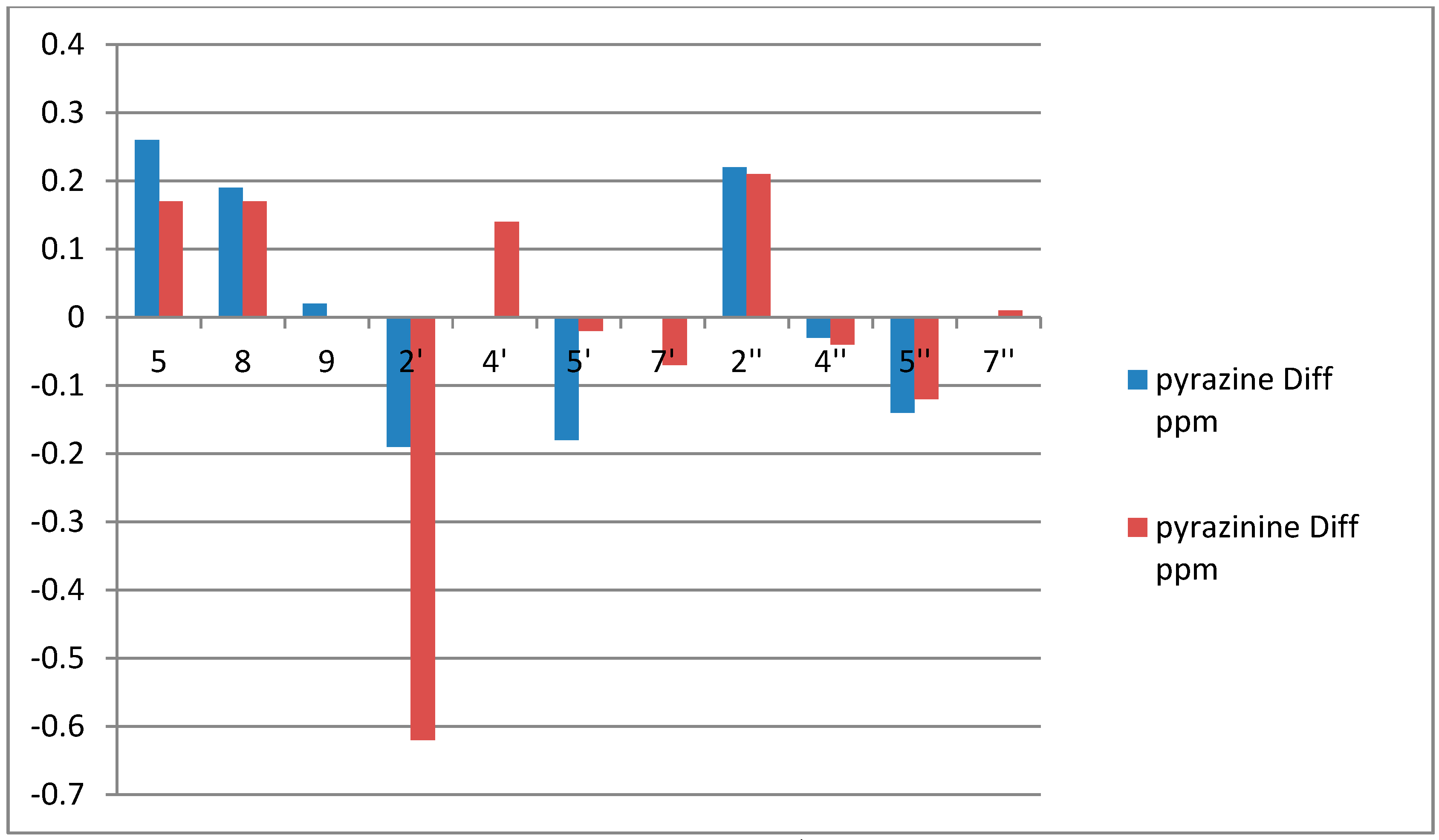 |
| (**c**) |

**Figure S28.** Calculated values for the pyrazine **1** versus pyrazinine structure **3** calculated using ACD ChemPredictor V.11 (Advanced Chemistry Development Toronto, ON, Canada). **(a)** Calculated values; (**b**) Differences between observed and calculated ^13^C Chemical shift data for **1** and **3**; (**c**) Differences between observed and calculated ^1^H Chemical shift data for **1** and **3**.
